# Glioblastoma Cells Induce Neuron Loss In Vivo and In Vitro

**DOI:** 10.3390/cancers17172817

**Published:** 2025-08-28

**Authors:** Komal N. Rawal, Charlotte Degorre, Philip J. Tofilon

**Affiliations:** Radiation Oncology Branch, National Cancer Institute, 10 Center Drive-MSC 1002, Building 10, B3B406, Bethesda, MD 20892, USA; charlotte.degorre@nih.gov (C.D.); philip.tofilon@nih.gov (P.J.T.)

**Keywords:** Glioblastoma, Glioblastoma stem cells, iPSC-derived Neuron Progenitor Cells, Neuron differentiation, GSC–neuron coculture, neuron death

## Abstract

**Background**: The vast majority of GBMs recur within 2 years following standard treatment, including radiotherapy. Seizures and epilepsy are common in GBM patients, suggesting tumor-cell-induced neuron toxicity. Additionally, the tumor cells and neurons interact during tumor development; however, the effects of tumor cells on the neurons remain unclear. **Methods**: Orthotopic xenografts initiated from GSCs expressing GFP implanted into the right striatum of nude mice were irradiated (10 Gy) 35 days after implantation, followed by immunohistochemistry (IHC) to investigate the tumor cell–neuron interactions. Moreover, we established a direct coculture of human GSCs and neurons differentiated from human iPSC-derived neural progenitor cells (NPCs) to investigate the impact of the tumor cells on the neurons. Neuronal cell counts were monitored to assess neurotoxicity. Culture CM were analyzed through cytokine profiling. **Results:** In untreated mice, tumors invaded across the right hemisphere (RH), with increased cell contact with the mouse neurons. In irradiated mice, the tumor regrowth was less invasive and had fewer neurons. In vitro, the GSCs induced neuronal death in the direct coculture. Similarly, the CM from the direct cocultures caused significant neuronal death. The cytokine analysis revealed that the cocultures uniquely secreted IL-8 into the CM. Furthermore, treatment with recombinant (r) human IL-8 caused significant neuron death, while IL-8 blocking antibodies prevented this neurotoxicity in the coculture. **Conclusions:** This study demonstrates that GBM tumors regrown after radiation lack neurons, and direct interaction between GSCs and the neurons is necessary for GSC-mediated neurotoxicity, likely involving IL-8 in neuronal death.

## Simple Summary

Glioblastoma (GBM) is the most aggressive brain tumor, with a high mortality rate even after the standard-of-care treatment. Seizures and epilepsy are common in about 60% of GBM patients, leading to neurological damage. GBM tumor cells interact with the neurons during tumor development and progression. Therefore, this study examined whether GBM tumors directly harm the neurons. In vivo studies demonstrated that tumors regrown post-radiation were less invasive but led to significant neuronal death within the tumor mass. To understand the effects of the tumor cells on the neurons, a direct coculture of neurons–glioblastoma stem cells (GSCs) was established, which showed that direct contact with the GSCs induced neuronal death. Notably, cytokine profiling identified that the neuron–GSC coculture produced interleukin (IL)-8 abundantly in conditioned media (CM). Furthermore, our study noted that IL-8 causes neuronal death, suggesting it could be a factor in mediating GSC-induced neuronal death. In conclusion, the study presented suggests that GSCs induce neuronal death.

## 1. Introduction

Glioblastoma (GBM) is a highly aggressive form of brain tumor, with a median survival rate of 12–16 months for GBM patients, even after the standard-of-care treatment, such as surgery, radiation, and chemotherapy [1,2,3]. About 90% of patients experience tumor recurrence, which is usually the mechanism of tumor progression. Seizures and epilepsy are major clinical manifestations observed in 60% of GBM patients, with episodes occurring in 30–62% of patients during this illness, leading to neurological disorders and neurotoxicity [4,5,6].

Studies have demonstrated that GBM cells interact with the neurons directly through tumor microtube formations and electrochemical synapses, and indirectly through secreted factors and extracellular vesicles [7,8,9]. Neuroglin-3, a synaptic connecting protein, activates the PI3K-mTOR signaling pathway, further upregulating several synaptic genes and associated neuronal activities, like neurotransmission and glutamate production. This hyperexcitability of the neurons impedes balance in neurotransmission, which promotes the growth, development, and recurrence of GBM tumors [10,11,12,13]. These events have been mapped using paired molecular signatures of tumors and neurons in peritumoral spaces [6,14]. Moreover, the GBM cells hijack neural mechanisms by integrating into neuronal circuits via several synaptic and electric connectomes, leading to neurotoxicity, GBM recurrence, and chemoradiotherapy resistance [15]. Tetzlaff et al. reported a sequence of events in GBM wherein GBM cell–neuron interactions are the primary steps, followed by hyperexcitability and subsequent neurological disorders [14]. GBMs are heterogeneous and have dynamic cellular complexity, with a high stemness index due to an abundant GSC population, to which tumor progression and resistance are attributed [16]. GSCs are characterized by self-renewal properties, high plasticity, and the ability to generate therapy-resistant tumors with DNA damage repair potential, a high mitochondrial reserve, and the ability to survive in a hypoxic niche. Therefore, GSCs are a valuable choice for understanding the development, progression, and recurrence of GBM [17,18,19]. They intervene in neuronal activities by hijacking the glutamatergic and GABAergic signaling pathways, which promote tumor cell invasion into the brain [8,20,21].

While most studies have focused on the association between neurons and GBM cells in tumor development and progression, less information is available on the repercussions of tumor cells on the fate of the neurons, leading to neurotoxicity.

To investigate the influence of GBM on neuronal death, we carried out this study using two models:

i. GSC-initiated orthotopic xenografts: In this model, human GSCs were implanted into the right striatum of mouse brains, and tumors were allowed to develop for 35 days. These tumor-bearing mice were irradiated at a dose of 10 Gy, and tumor regrowth was monitored for up to 21 days post-irradiation. It was observed that the non-irradiated tumors diffusively spanned the RH and the olfactory bulb (OB), where tumor cells interacted with the neurons. Conversely, the irradiated tumor-bearing mice exhibited subsequent tumor mass regrowth with concomitant depletion of the neurons. This study highlights the regrowth of GBM following irradiation, which causes substantial damage to the neurons.
ii. An in vitro direct coculture model of GSCs and human neurons: To further understand the crosstalk between the GSCs and neurons, we established an in vitro direct coculture model using GSCs and human neurons differentiated from iPSC-derived NPCs. This approach, using both cell types derived from humans, provides an advantage for studying the interaction of GBM tumor cells and neurons in GBM, thereby replicating the actual interaction between these two cell types in GBM patients. Moreover, this model is easy to use for cellular assays in scrutinizing the effects of GSCs on neuronal death. Of note, the in vitro studies demonstrate that GSCs have detrimental effects on the neurons. We identified IL-8 as a potential cytokine secreted as a result of the GSC and neuron coculture, which could be a potential candidate for further investigations into GBM’s development and recurrence.

## 2. Materials and Methods

### 2.1. Cells

#### 2.1.1. Glioblastoma Stem Cells

Two types of GSCs, NSC11 and NSC20, isolated from surgical specimens of human GBM were provided as frozen stocks by Dr. Frederick Lang (MD Anderson Brain Tumor research program, The University of Texas MD Anderson Moon Shots Program^TM^, and the Broach Foundation for Brain Cancer Research, Houston, TX, USA). The cells were grown as suspension cultures in the form of neurospheres in the GSC media, which consisted of DMEM/F-12 (Invitrogen, Waltham, MA, USA), B27 supplement (Invitrogen), EGF, and human r-fibroblast growth factor (FGF) 50 ng/mL each, (R&D Systems, Minneapolis, MN, USA) [22]. The cells were cultured under culture conditions of 5% O_2_, 5% CO_2_, and 37 °C. These cells were sorted for the CD133^+^ population, and both types of GSCs expressed eGFP2 and the bioluminescent Luciferase enzyme ffLuc2 under the control of the Ubc promoter (LVpFUGQ-UbC-ffLuc2-eGFP2) [23]. Once revived, the cells were used as young cultures within 2 months, followed by thawing new cells to conduct further experiments.

#### 2.1.2. Neural Progenitor Cells

iPSC-derived NPCs (Catalog #ACS5004) were procured from the American Type Culture Collection (ATCC, Manassas, VA, USA). They were revived in ENStem-A Neural Expansion Medium (Sigma Aldrich SCM004, Burlington, MA, USA) supplemented with 2 mM of glutamine and 100 µg/mL of FGF-2. The cells were seeded at a density of 80,000 cells/cm^2^ onto tissue culture plates coated with Cell Matrix Basement Membrane Gel (ATCC ACS-3035). The cells were grown in 5% O_2_ and 5% CO_2_ at 37 °C until they reached 95% confluence. The cells were used within 10 passages for all experiments.

#### 2.1.3. The MRC9 Fibroblast Cell Line

The MRC9 fibroblast cell line, derived from the lungs of a normal healthy male, was procured from the ATCC (CCL-212). The cells were grown in DMEM supplemented with 10% FBS (Gibco, Newyork, NY, USA), glutamine (2 mM), Sodium Pyruvate (1 mM), and non-essential amino acids (1×) in the standard 20% O_2_, 5% CO_2_, 37 °C culture conditions. For the coculture experiments, the cells were cultured in GSC media, and the same culture conditions as those for the GSCs were used. The cells were used within 10 passages for all experiments.

### 2.2. The Orthotopic Xenograft Mouse Model

A total of 1 × 10^5^ NSC11 cells were implanted into the right striata of 6-week-old female nude mice (NCr nu/nu; NCI Animal Production Program). The tumor growth was monitored through bioluminescence imaging (BLI), as previously described, with the tumors allowed to grow for 35 days. Mice were randomized according to BLI total flux and grouped into non-irradiated (0 Gy) and irradiated groups (10 Gy), each consisting of 4–5 mice. Irradiation was performed as previously described [23]. Briefly, the mice were anesthetized with a ketamine/xylazine cocktail and positioned into plexiglass jigs for X-ray irradiation with a 10 Gy dose. The tumor regrowth was assessed at 14 days and 21 days post-irradiation. All experiments were conducted as per the principles and procedures approved by the NIH Guide for Care and Use of Animals and following the approval of the Institutional Animal Care and Use Committee.

### 2.3. Neuron Differentiation

NPCs were seeded at a density of 10,000 cells/cm^2^ in NPC media onto precoated tissue culture plates. The next day, the media was replaced with neuronal differentiation media (SCM004, Sigma). The media was replaced every 2–3 days for 3 weeks until the NPCs had differentiated into neurons. The neurons were grown under the same culture conditions as those for the GSCs. Immunocytochemistry (ICC) was employed to characterize the neurons. Differentiated neurons were used for all of the experiments in this study. The neurons for the GSC–neuron coculture experiments were cultured in GSC media.

### 2.4. Establishment of the GSC–Neuron Coculture

NSC11 or NSC20 cells were directly seeded at a ratio of 1:10 cells on differentiated neurons into the GSC media. The cocultures were characterized by the presence of NeuN-positive neuronal cells, and GFP^+^ GSCs were observed through ICC. The cocultures were maintained in GSC media for up to 96 h for specific experiments.

### 2.5. The Cell Count Assay

For the coculture experiments, the neurons, GSCs, and MRC9 cells were counted using a hemocytometer in a BioTek Lionheart FX Automated Microscope (Agilent, Santa Clara, CA, USA). Both non-GFP cells (neurons or MRC9) and GFP-expressing cells (NSC11 and NSC20) were imaged simultaneously in the same field using the GFP (Alexa Fluor (AF488)) and phase contrast filters. The cells were counted and normalized to the control neurons or GSCs, respectively. Additionally, the cells were counted on a Countess Cell Counter for the regular cell culture and other experiments.

### 2.6. Immunohistochemistry

Brain IHC was adapted from our previously reported protocol [23]. Briefly, the mice were euthanized at specific experimental time points via ketamine/xylazine injections, followed by cervical dislocation. Cardiac perfusion was performed with PBS and then with 10% formalin. Their whole brains were subsequently removed and stored in 10% formalin, followed by PBS after 48 h. The brain tissues were cut into 10 µm thick sections. Xylene-mediated dehydration and subsequent rehydration steps with a series of ethanol concentrations were performed. The sections were blocked using blocking solution (PBS containing 10% FBS, 1% BSA, and 0.3% Triton-X). Primary antibodies against NeuN and GFP were applied to the tissues, and they were incubated overnight at 4 °C. The following day, the tissue sections were washed with PBST and incubated with the secondary antibodies donkey anti-rabbitAF647 and donkey anti-goat-AF488 for 1 h at room temperature (RT). The sections were then washed with PBST, stained with DAPI, and mounted with anti-fade solution. The slides were imaged using a Zeiss Axio Scan 7 Scanner, (Carl Zeiss, Oberkochen, Germany) with a 40× magnification lens. The images were analyzed with Zen 2.3 Blue software. The details of the antibodies used in this study are described in Table 1.

### 2.7. Immunocytochemistry

After the experiments, the cells were fixed with 10% formalin solution, followed by permeabilization with PBS containing 0.2% Triton-X. The cells were then blocked (using PBS, 1% BSA, 5% FBS, and 0.25% Tween-20). The NeuN, Tuj1 (B-III Tubulin), GFP, and ƴH2AX primary antibodies were prepared in PBST with 1% BSA and were added and incubated at 4 °C. The next day, the cells were incubated with the secondary antibodies conjugated with AF488 and AF647, along with 1 µg/mL DAPI, for 1 h at RT and mounted using Prolong Diamond Antifade (Invitrogen). Table 1 describes the details of all of the antibodies used. The images were captured on an Apotome Axio A3 fluorescent microscope, Carl Zeiss.

DNA damage was monitored in the cocultures at intervals of 1 h, 6 h, and 16 h after 3 Gy irradiation. The cells were fixed and stained with a ƴH2AX antibody, and then images were captured using Z-stacks on an LSM 780 confocal microscope, Carl Zeiss, with a 40× oil immersion objective lens. The images were analyzed in orthogonal projections, and the ƴH2AX foci were counted in each group and analyzed further.

### 2.8. The Western Blot Analysis

The cells were lysed and proteins were extracted using Radioimmunoprecipitation Assay (RIPA) buffer supplemented with 1× HALT protease inhibitor cocktail (Thermo Scientific, Waltham, MA, USA) and 1× phosphatase inhibitor cocktails II and III (Sigma-Aldrich). The cells were sonicated at 60% amplitude with a 30 s pulse on/off cycle for 3 min. The cell lysates were incubated on ice for 30 min, followed by centrifugation at 12,000 rpm for 30 min at 4 °C. Total protein quantification was carried out through a BCA assay (Thermo Scientific), and 40 µg of protein was separated on 4–20% SDS-PAGE gel and then transferred onto a nitrocellulose membrane (Biorad, San Francisco, CA, USA). The proteins were blocked with 5% non-fat dry milk (Biorad) blocking solution for 1 h at RT, and the PARP or actin antibodies were probed overnight at 4 °C, after which point further incubation with the secondary antibodies was conducted for 1 h at RT. The details on the antibodies are mentioned in Table 1. Protein bands were captured using Pierce ECL Western blotting substrate (Thermo Scientific) and the ChemiDoc MP imaging system.

### 2.9. The Human Cytokine Array

The culture CM of neurons, NSC11 and NSC20 cells, and cocultures (after 48 h (h) of coculture) were subjected to a cytokine profile analysis using the Raybiotech -AAH-CYT-5-8 kit (Raybiotech, Peachtree Corners, GA, USA) according to the manufacturer’s instructions. Briefly, the CM were collected and centrifuged at 4000 rpm for 15 min at 4 °C to remove cell debris. The supernatants were immediately stored in a −80 °C deep freezer until further use. Upon thawing, the media underwent a second centrifugation at 10,000 rpm for 10 min. Finally, the culture supernatants were used for the cytokine analysis. The array membranes were incubated with blocking solution for 30 min at RT. Individual samples were then added to each array membrane and incubated overnight at 4 °C. The next day, the membranes were washed and subsequently coupled with a biotinylated antibody, followed by an HRP–streptavidin antibody for 2 h each. The membranes were then exposed to the detection buffer cocktails, and cytokine spots were visualized via chemiluminescence. The spots were analyzed using ImageJ 1.54g, and the background was subtracted from the signal data. The signal intensities for each cytokine were determined after subtracting the negative control values and then dividing by the positive control values. The resultant signal intensities were normalized to those for the control GSC media.

### 2.10. The IL-8 Cytokine Treatment

The neurons were treated with r-human IL-8/CXCL8 protein (208-IL-010, R&D Systems), with a concentration range of 10 ng/mL to 100 ng/mL, for 72 h. Neuron cell death was monitored every 24 h by taking the cell count.

### 2.11. The IL-8-Neutralizing Antibody Treatment

The cocultures of the NSC11/NSC20 cells with the neurons after 48 h of incubation were treated with the IL-8-neutralizing antibody (807) AB18672 at a concentration of 0.5 µg/mL, Abcam, for 48 h to assess the effects of IL-8 on neuronal death in the coculture. The neutralizing effect of the IL-8 antibody was evaluated by taking the cell count according to the method explained in Section 2.5.

### 2.12. The Statistical Analysis

All experiments were performed with three independent experimental setups, and the results are presented as the mean ± S.E.M. Statistical significance was calculated using the two-tailed Student’s *T*-test, where significance is indicated by * *p* < 0.05. GraphPad Prism version 10.1.1 was used for the statistical analysis.

## 3. Results

### 3.1. Tumors That Regrow Post-Irradiation Are Devoid of Neurons

**Figure 1:**
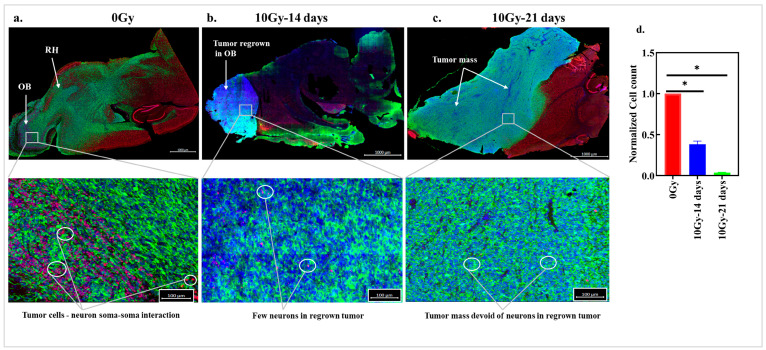
Tumors that regrow post-irradiation are devoid of neurons: representative IHC images of 40× scans of right sagittal sections of the brains from NSC11-initiated-tumor-bearing mice, treated with 0 Gy and irradiated with 10 Gy 35 days post i.c. implantation. Cell nuclei were stained using DAPI (blue), GFP-expressing tumor cells were stained using GFP (green), and NeuN-expressing neurons were stained with the NeuN Ab (red). (**a**) IHC of sagittal sections of mouse brains treated with 0 Gy showing the interaction between GFP^+^ tumor cells and NeuN^+^ mouse neurons; scale bar: 1000 µm. The lower panel image is a 35% magnified version of (**a**); scale bar: 100 µm. (**b**) IHC of brain sections with tumor regrowth 14 days post 10 Gy irradiation; scale bar: 1000 µm. The magnified image shows a tumor mass with reduced neurons. The lower panel image is a 35% magnified version of (**b**); scale bar: 100 µm (**b**), (**c**) Histology of the tumor mass 21 days post-irradiation; scale bar: 1000 µm. The magnified image shows that there are no neurons in the tumor mass regrown after 35 days of radiation. The image is 35% magnified; image scale bar: 100 µm. (**d**) Neuron counts are represented as the mean of the normalized neuron count ± the S.E.M. of three different mice in each group. *p* value * < 0.05. OB: olfactory bulb; RH: right hemisphere.

To evaluate the effects of GBM tumor cells on neurons, human GFP-expressing GSCs (NSC11) were implanted into the right striata of nude mice. Sagittal sections of the right sides of the brains from the untreated (0 Gy) mice underwent IHC for GFP and NeuN staining (Figure 1a, Document S1) to illustrate the GSCs and the soma of the mouse neurons, respectively. Staining revealed that the GFP-expressing NSC11 cells migrated from the RH and invaded the OB, forming diffuse tumors. NeuN-positive neurons were present throughout the RH and OB, having cell-to-cell contact with the GFP^+^ NSC11 cells. Because radiation is part of the standard of care for GBM [24,25], the tumor cell–neuron contact was evaluated in tumors that regrew after irradiation. NSC11 cells were intracranially implanted into the brains of the mice, and tumor development was monitored for 35 days. The tumor-bearing mice were irradiated with a 10 Gy dose, followed by tumor collection after 14 days and 21 days for IHC studies. As shown in Figure 1b, the tumors that regrew were less diffused and primarily regrew as a mass confined to the OB area. This is consistent with previous work in this model system, showing that tumors that migrate to the OB are radioresistant [23,26]. Immunostaining revealed that there were few neurons within the tumor mass at 14 days. By 21 days, a recurrent tumor occupied a major portion of the brain and the OB, where neurons were not detected in the tumor mass or in the well-demarcated border between the tumor and normal tissue (Figure 1c). For tumor cells that escaped the tumor mass, there was detectable contact with the neurons in the RH. Figure 1d demonstrates that there was a significant loss of neurons on day 14 and day 21 post-irradiation in the regrowing tumor mass compared to that in the tumor-bearing control mice irradiated with 0 Gy. These results demonstrate that the tumor mass that regrows after radiation lacks neurons.

### 3.2. The Effect of Radiation on the Neuron Distribution in Mice Without Tumors

**Figure 2:**
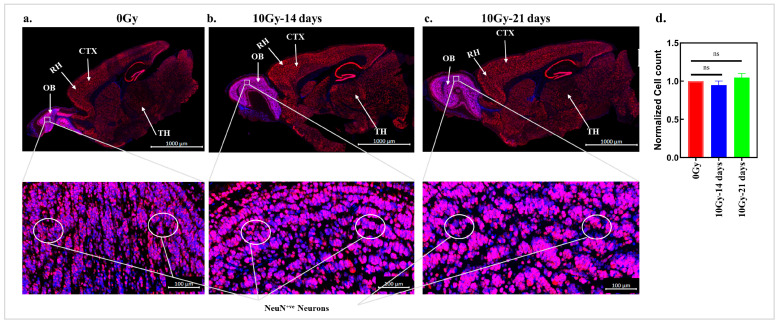
The effect of radiation on the neuron distribution in mice without tumors: representative IHC images of 40× magnified sagittal sections of the brains from mice without tumors. DAPI (blue) was used to stain cell nuclei, and neurons were stained for NeuN (red). (**a**) IHC of a sagittal section of the brain from a 0 Gy-irradiated mouse. Scale bar: 1000 µm. The magnified image shows the distribution of the neurons throughout the brain. The image in the lower panel is 35% magnified; image scale bar: 100 µm. (**b**) IHC results for a brain section from a mouse after 14 days of 10 Gy irradiation. scale bar: 1000 µm. The image in the lower panel is 35% magnified, demonstrating no effect of radiation on the neurons; image scale bar: 100 µm. (**c**) IHC results for a brain section 21 days post-radiation; scale bar: 1000 µm. The magnified image depicts the presence of neurons throughout the brain section. The magnified image is a 35% magnified version of the section in (**c**); image scale bar: 100 µm. (**d**) Neuron counts are represented as the mean of the normalized neuron count ± the S.E.M. of three separate mice. *p* value: ns > 0.05 (non-significant). OB: olfactory bulb; RH: right hemisphere; CTX: cerebral cortex; TH: thalamus.

Because neuron loss was only observed in the tumor-bearing mice after irradiation, we assessed the effects of radiation on the neuron frequency and distribution in the non-tumor-bearing mice. Mice without brain tumors received 10 Gy, and the sagittal sections of the right side of the brains from both the untreated and 10 Gy-treated mice were examined for NeuN-positive neurons at 14 and 21 days post-irradiation. Histologically, there were no differences in the brain morphology between the control and irradiated mice. In untreated mice, neurons were distributed throughout the brain (Figure 2a). Examination of the brain sections after 10 Gy revealed no gross changes in the neuron distribution (Figure 2b,c), unlike those seen in the mice with tumor regrowth after irradiation (Figure 1b,c). Moreover, NeuN-expressing neurons were present in various regions of the brain, like the cerebral cortex (CTX), the thalamus (TH), and parts of the main and anterior OB at a high density. The magnified images of the brain sections from the 0 Gy- and 10 Gy-irradiated mice shown in the boxes show a dense NeuN-positive neuronal population throughout the OB in both groups of mice. Figure 2d depicts that there was no significant difference in the neuron count in the irradiated mice compared to that in the control normal mice. While neuron function was not assessed, these data suggest that radiation alone does not induce neuron loss.

### 3.3. GSCs Lead to the Loss of Neurons in the Direct Coculture

**Figure 3:**
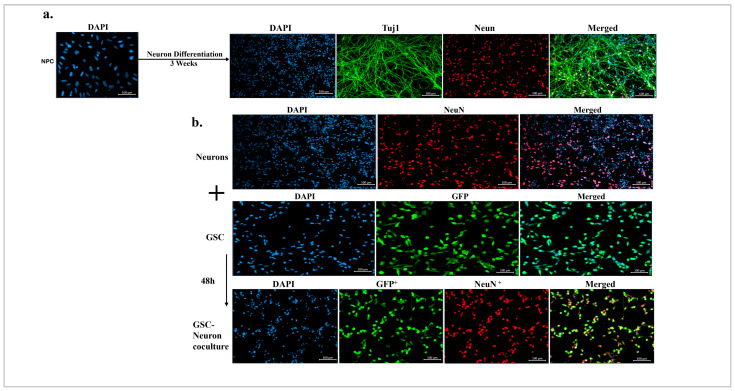
Establishment of the GSC–neuron coculture. (**a**) Neuron differentiation: iPSC-derived NPCs were induced to differentiate into neurons for three weeks and were characterized for the neuronal markers Tuj1 (green) and NeuN (red). Cell nuclei were stained with DAPI (blue). (**b**) Establishment of the GSC–neuron coculture: NeuN^+^ neurons were used for the cocultures. GFP^+^ GSCs (NSC11 and NSC20) were added directly to the neurons in the GSC media for 48 h to establish the coculture. The figure shows representative images of the GSCs (GFP: green)–neurons (NeuN: red) in the direct coculture. The images were captured with a confocal microscope using a 40× lens, and images of the neurons are presented as orthogonal projections.

To better understand the neuron loss detected in the in vivo studies in Figure 1, we used an in vitro coculture model comprising GSCs (NSC11 and NSC20) expressing GFP and human iPSC-derived NPCs. In this model, NPCs were induced to differentiate into neurons and were characterized by the presence of Tuj1 and NeuN, as shown in Figure 3a. GSCs were directly added to the neuronal monolayers, and both cell types were allowed to interact for 48 h to establish a coculture (Figure 3b). The images shown in the figure are representative of the NSC11 and NSC20 cells used in the coculture models. The cocultures were stained for GFP and NeuN to identify GFP-expressing GSCs and the soma of NeuN^+^ neurons, respectively.

**Figure 4:**
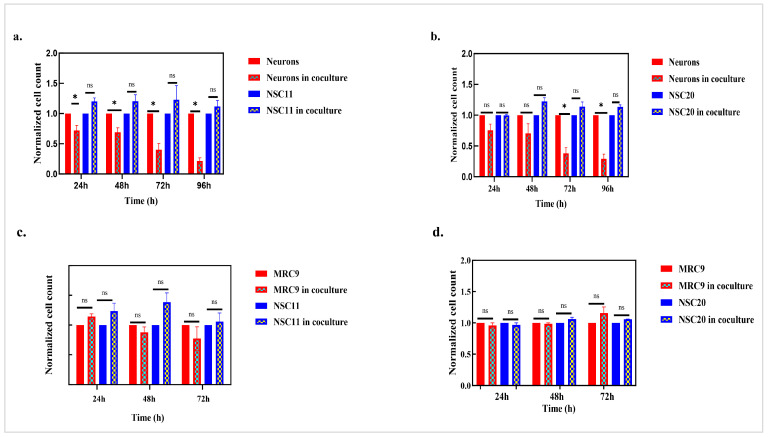
GSCs lead to neuron loss in direct coculture: GSCs were directly cocultured with neurons. (**a**) Cell counts of neurons, neurons in the cocultures, NSC11 cells, and NSC11 cells in the cocultures were taken after every 24 h in the coculture up to 96 h. *p* value: ns > 0.05: non-significant. (**b**) Cell counts of neurons, neurons in the cocultures, NSC20 cells, and NSC20 cells in the cocultures were measured at intervals of 24 h until 96 h post-coculture. The results are presented as the mean cell count normalized to the respective neurons or GSCs ± S.E.M. for three independent experiments. *p* value * < 0.05, *p* value: ns > 0.05 (non-significant). (**c**) GSCs do not affect normal fibroblast cells. MRC9 cells were cocultured with NSC11 cells for 72 h, and the cell count of both cell types, individually cultured or in coculture, was taken at an interval of 24 h up to 72 h. The results are presented as the mean normalized cell count ± S.E.M. for *n* = 3. *p* value: ns > 0.05 (non-significant). (**d**) MRC9 cells were cocultured with NSC20 cells for 72 h, and the cell count of both cell types, individually cultured or in coculture, was taken at an interval of 24 h up to 72 h. The results are presented as the mean normalized cell count ± S.E.M for 3 different experiments. *p* value: ns > 0.05 (non-significant).

To determine the effects of the coculture on the survival of each cell type, initially, the number of neurons and GSCs was determined as a function of time in the coculture. As shown in Figure 4a, the neuron numbers decreased after the addition of the NSC11 GSCs, reaching the maximum decrease by 72 h in the coculture. However, no significant difference was found in the number of NSC11 cells. A similar loss of neurons was detected when the NSC20 cells were cocultured with the neurons (Figure 4b). These results suggest that GSCs induce neuron death in vitro, consistent with the findings in vivo.

To determine whether the GSC-induced cell loss was specific to neurons, the consequences of a coculture with human normal fibroblasts (MRC9) were evaluated (Figure 4c,d). MRC9 cells were seeded at an approximately 60–70% density and were allowed to settle overnight. The next day, GSCs were seeded onto the fibroblast monolayer. This coculture setup was also grown in GSC media and 5% CO_2_ and 5% O_2_ culture conditions, similar to those for the GSC–neuron cocultures. The cell counts of both the MRC9 and NSC11 cells were noted in the cocultures and monolayers from 24 h to 72 h. Figure 4c shows that there was an increase in the NSC11 cells at 24 and 48 h, but no significant difference in the MRC9 cell count was detected when they were cocultured with the NSC11 cells for up to 72 h. For the NSC20-MRC9 cocultures, no change in NSC20 was detected, and as for NSC11, no loss of MRC9 cells was detected for up to 72 h (Figure 4d). These results show that GSCs are not toxic to all cell types.

**Figure 5:**
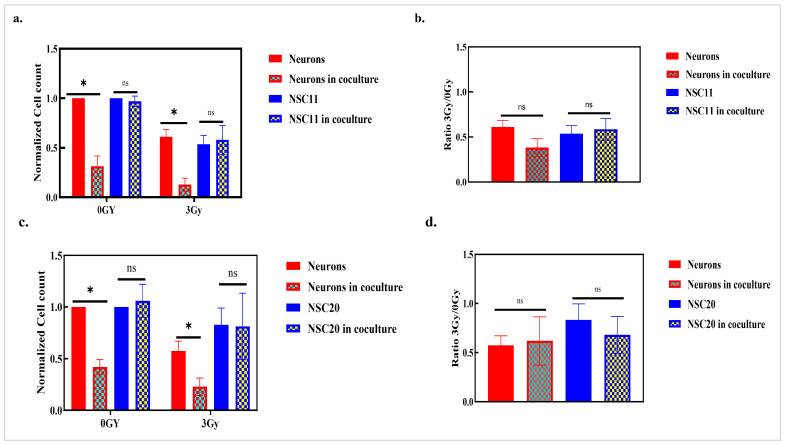
The effect of radiation on neuronal death. To examine the additive effects of irradiation on neuronal death, (**a**) cocultures of the neurons and NSC11 cells were subjected to 3 Gy irradiation after 48 h of incubation, and the cell counts were taken after 48 h of incubation in both the 0 Gy and 3 Gy groups. The results are presented as the mean normalized cell counts ± the S.E.M. of three different experiments. *p* value * < 0.05. *p* value: ns > 0.05 (non-significant). (**b**) The ratio of neurons, neurons in the coculture with NSC11 cells, NSC11 cells, and NSC11 cells in the coculture under 3 Gy/0 Gy has been presented. The results are represented as the ratio of the 3 Gy/0 Gy cell count ± the S.E.M. of three independent experiments. *p* value: ns > 0.05 (non-significant). (**c**) Normalized cell counts for all groups and non-radiated and radiated cocultures with the NSC20 cells are presented. The results are presented as the mean normalized cell counts ± the S.E.M. of three different experiments. *p* value * < 0.05. *p* value: ns > 0.05 (non-significant). (**d**) A graph representing the 3 Gy/0 Gy ratio for all cell types is presented. The results are presented as the mean normalized cell counts ± the S.E.M. of three different experiments. *p* value: ns > 0.05 (non-significant).

Because the in vivo experiments described in Figure 1 revealed neuron loss only in the irradiated tumor-bearing mice, we evaluated the impacts of radiation on an in vitro GSC–neuron coculture model. The cells were irradiated (3 Gy) after 48 h of coculture. The number of each cell type was determined 48 h post-irradiation. The results showed that in the cocultures with either the NSC11 or NSC20 cells, the neurons were depleted in the 0 Gy groups. Following exposure to 3 Gy, the neuron counts were depleted further. However, the survival ratios for the neurons (3 Gy to 0 Gy) indicated no significant differences (Figure 5a–d). These results suggest that irradiation does not enhance the neurotoxic effects exerted by GSCs in cocultures.

Furthermore, to support our findings, we evaluated the effects of irradiation on DNA double-strand break repairs. The cocultures were exposed to 3 Gy irradiation and were evaluated for ƴH2AX foci at intervals of 1 h, 6 h, and 16 h post-irradiation. Figure S1a is a representative confocal image of the ƴH2AX foci in the direct GSC–neuron cocultures after 1 h of 3 Gy exposure, wherein the GSCs express GFP (green), and the neurons are only stained with DAPI (blue), while the ƴH2AX foci appear red. The results elucidated that the neurons had the highest ƴH2AX foci count after 1 h of irradiation, which gradually decreased at 16 h, with no significant difference in the foci count in the neurons cocultured with NSC11 or NSC20 cells compared to that in the controls. Interestingly, on the other hand, both the NSC11 and NSC20 cells cocultured with the neurons had significantly lower ƴH2AX foci counts compared to their controls, and the cells had recovered from DNA damage at around 16 h of irradiation (Figure S1b,c). These results support our observation that irradiation does not enhance the neurotoxic effects of GSC-induced neuronal death further. However, the GSCs present in direct proximity to the neurons in the cocultures had an enhanced capacity to repair radiation-induced DNA damage.

### 3.4. Cell–Cell Contact Is Required for GSC-Mediated Neurotoxicity

**Figure 6:**
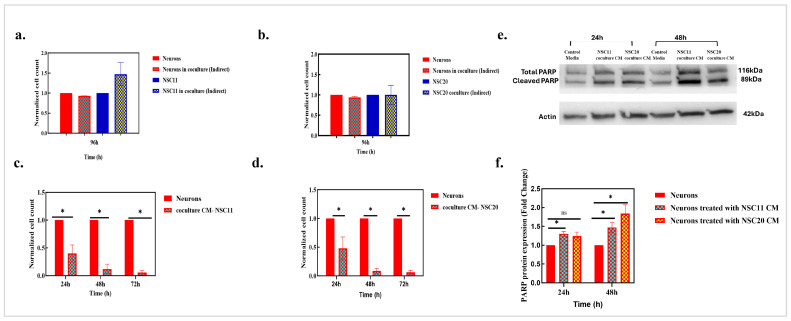
Cell–cell contact is required for GSC-mediated neurotoxicity. (**a**) Indirect coculture does not have any effect on neuronal death. NSC11 cells were cultured on cell inserts, which were then placed onto neurons for an indirect coculture for 96 h and were subjected to cell counting. The results are presented as the mean of the normalized cells ± the S.E.M. from 3 independent experiments. *p* value: ns > 0.05 (non-significant). (**b**) NSC20 cells were cocultured indirectly with the neurons for 96 h. The cell counts were recorded, and a graph was plotted with the mean of the normalized cells ± the S.E.M. of 3 independent experiments. *p* value: ns > 0.05 (non-significant). (**c**) Coculture CM are toxic to neurons. Neurons were treated with the coculture CM of NSC11 cells, followed by estimating the surviving neurons every 24 h for up to 72 h. The results are presented as the mean normalized cell count compared to the control neuron cells ± the S.E.M. of three independent experiments. *p* value * < 0.05. (**d**) Neurons were treated with coculture CM of NSC20 cells for 72 h, and cell counts were taken every 24 h for up to 72 h. The results are presented as the mean normalized cell count compared to that in the control neuron cells ± the S.E.M. of three independent experiments. *p* value * < 0.05. (**e**) Coculture CM induce neuronal cell death. Representative Western blot results for PARP showing total PARP at 116 kDa and cleaved PARP at 89 kDa. The internal control used was actin, as shown at 42 kDa. The blot image has been horizontally flipped for the proper order orientation. (**f**) The densitometric analysis of the protein expression of PARP normalized to that in the control neurons. The results are represented as the mean fold change in PARP protein expression ± the S.E.M. of three independent experiments. *p* value * < 0.05. *p* value: ns > 0.05 (non-significant).

In the coculture model used in Figure 4, the GCSs were seeded directly onto the neurons. To determine whether direct cell–cell contact was required for neuronal toxicity, we used an indirect coculture system. In this setup, GSCs were seeded onto cell inserts and placed on top of the neurons. Only soluble components of the GSC media were exchanged between the two cell types, creating indirect coculture conditions. While there was an increase in the number of NSC11 cells under these coculture conditions, no change in the neurons was detected. For the NSC20 cell–neuron cocultures, there was no significant change in the number of GSCs or neurons (Figure 6a,b). These results suggest that GSCs do not induce neuronal loss when cultured indirectly, emphasizing that cell–cell contact is necessary for GSC-induced neuron toxicity. To characterize the GSC-induced neuron loss detected in the direct coculture model further, the coculture CM were evaluated for neurotoxic effects. The CM were collected after 48 h of GSC–neuron coculture and were directly added to the neurons. As shown in Figure 6c,d, the addition of the coculture CM to the neuron cultures resulted in a significant loss of neurons by 24 h, with a greater than 90% loss by 72 h.

The coculture-CM-treated neurons were examined further for the expression of PARP-1, which is an important marker of cell death. Coculture-CM-induced cell death in the neurons was assessed through a Western blot analysis of the PARP-1 protein expression. The neurons treated with the CM from the NSC11 cell–neuron or NSC20 cell–neuron cocultures showed significant expression of both total and cleaved PARP-1 protein after 24 h and 48 h of treatment, as shown in Figure 6e. The upregulation of both forms of PARP-1 indicates activity in the neurons, as they are susceptible to oxidative-stress-mediated DNA damage. However, it can lead to neuronal death under neurotoxic conditions [27]. Additionally, PARP also plays an essential role in neuron differentiation; therefore, it is detected in neuronal cells [28], and a densitometric analysis was performed on both bands of the PARP-1 protein, as shown in Figure 6f. Actin served as an internal control. The results show that direct interaction between GSCs and neurons in a coculture model leads to the secretion of some factors from the secretome/the CM of the direct coculture which are toxic to the neurons.

### 3.5. GSC–Neuron Direct Coculture Secretes Cytokines That Influence Neuronal Death

**Figure 7:**
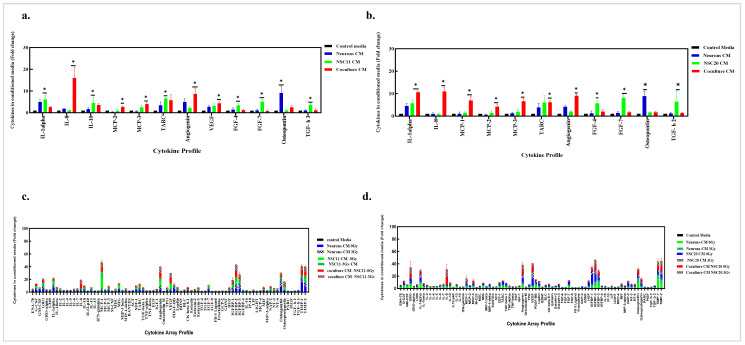
GSC–neuron direct coculture secretes cytokines that influence neuronal death. The profiles of 80 cytokines in the culture CM for the neurons, GSCs, and cocultures were evaluated 48 h post-coculture. GSC media were used as the control media. (**a**) The graph represents significantly differentially expressed cytokines from the CM with the neurons, NSC11 cells, and NSC11–neuron cocultures. The results are presented as the mean fold change in the cytokines ± the S.E.M. of three independent experiments. *p* value * < 0.05. (**b**) The cytokine profiles of significantly differentially expressed cytokines in the NSC20 cell–neuron coculture. The graph represents the mean fold change in the cytokines normalized to that in the control GSC media ± the S.E.M. of 3 different experiments. *p* value * < 0.05. (**c**) Cytokine profiles of CM media from NSC11 cell culture and the coculture after 48 h of 3 Gy radiation. The results of 2 separate experiments are presented as the mean fold change in the cytokines ± the S.E.M. (**d**) The graph represents the profiles of 80 cytokines from the cocultures of the NSC20 cells and neurons after 48 h of 3 Gy radiation, presented as the mean fold change in the cytokines ± the S.E.M. of 2 independent experiments.

As an initial investigation into the mechanisms mediating neuronal death in the GSC coculture, cytokine profiling was performed on the coculture CM. All of the cytokine levels detected in the CM were normalized to those in the control GSC media (Figure S2). The profiles for all 80 cytokines are shown in Figure S3. Differentially expressed cytokines in the CM of cocultures versus those in the neurons and GSCs alone are shown in Figure 7a,b. These figures illustrate that the neurons secrete IL-1α and angiogenin and uniquely express osteopontin. Moreover, in the CM from both the NSC11 and NSC20 cultures, IL-1α and TARC were secreted, and FGF-4, FGF-7, and TGF-β were distinctly expressed. In the coculture CM from both, the GSCs secreted the monocyte chemoattractant protein (MCP (MCP-1, MCP-2, MCP-3)) family and angiogenin, with the largest increase observed in IL-8. These results indicate that the interaction between the neurons and GSCs leads to enhanced IL-8 secretion in the coculture CM. Further, the effects of radiation on the cytokine profiles were examined. Samples were collected after 48 h of 3 Gy irradiation, and the cytokine profiles were found to resemble those in their respective control cultures. (Figure 7c,d). These results support the observation that irradiation does not contribute additionally to neurotoxicity.

### 3.6. The Effect of IL-8 on Neuronal Death

**Figure 8:**
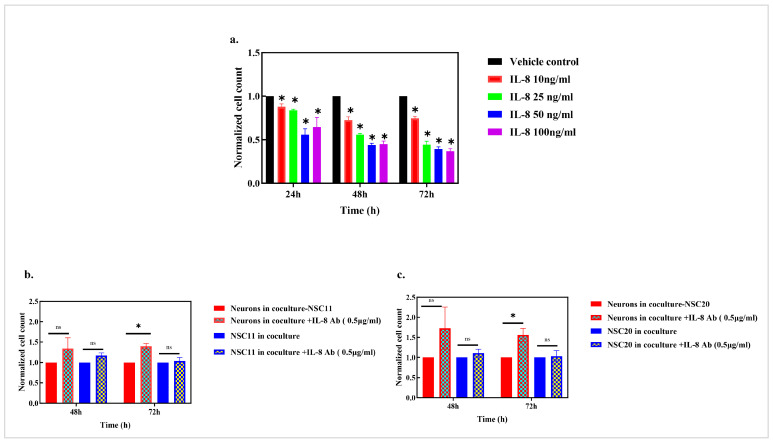
(**a**) The effects of IL-8 on neurons. The effect of the rIL-8 protein on neurotoxicity: neurons were treated with the rIL-8 protein within a concentration range of 10 ng/mL to 100 ng/mL for 72 h. Neuronal toxicity was determined using the cell count every 24 h up to 72 h. The graph represents the mean of the normalized cell count of the neurons ± the S.E.M. *p* value * < 0.05. (**b**) The IL-8-neutralizing Ab rescues neurons. Neurons and NSC11 cells in the cocultures treated with the IL-8 Ab were counted after 48 h and 72 h. The results represent the mean of the normalized cell count ± the S.E.M. of 3 different experiments. *p* value * < 0.05. *p* value: ns > 0.05 (non-significant). (**c**) Cell counts of neurons, neurons in the coculture with NSC20 cells, NSC20 cells alone, and NSC20 cells in the coculture. Results represented as the normalized cell count ± the S.E.M. of three separate experiments. *p* value * < 0.05. *p* value: ns > 0.05 (non-significant).

Of the cytokines, the GSC–neuron coculture led to the largest increase in the secretion of IL-8. As shown in Figure 8a, the addition of IL-8 to the neuron cultures resulted in a time- and dose-dependent loss of neurons. To evaluate the role of IL-8 in neuron loss further, an IL-8-neutralizing antibody was added to the GSC–neuron cocultures. In contrast to the loss of neurons in the GSC–neuron coculture, as shown in Figure 4, the addition of the IL8-neutralizing antibody resulted in an increased number of neurons in the NSC11 and NSC20 cocultures (Figure 8b,c). These results indicate that the IL-8-neutralizing antibody rescued the neurons in the cocultures with the GSCs, suggesting that IL-8 contributes to neuron loss when cocultured with GSCs.

## 4. Discussion

GBM patients suffer from seizures and epilepsy-associated neurological disorders because of tumor-mediated neurotoxicity. Significant efforts have been made to investigate the interactions between tumor cells and neuronal networks during tumor progression and recurrence [12,14,21,29,30], but there is limited information on the direct effect of GBM tumor cells on the fate of the neurons. In the present study, in non-irradiated tumor-bearing mice, the tumor cells had cell-to-cell contact with the neurons, supporting studies that report GBM cells invade the brain through direct interaction with the neurons and the neuronal network for tumor progression and development [11,12,13]. These observations align with the notion that GBM tumors tend to migrate toward the neurogenic regions of the OB and the subventricular zone [31,32]. Moreover, we reported that the GBM tumor cells become more radioresistant in the OB, with an increased DNA repair capacity [23,26]. In contrast, after 10 Gy radiation, recurrent tumors were confined to the OB region and were devoid of neurons. However, the role of radiation in neuronal loss in recurrent tumors is unclear in that irradiation of mice without tumors has no apparent effect on neuron distribution. These initial findings therefore led us to investigate the effects of GBM tumor cells on the neurons further using an in vitro coculture model.

In this model system, tumor cells were represented by GSCs, which were used for the initial tumor implants in the in vivo experiments. Neurons were generated from human iPSC-derived NPCs, which allowed for the evaluation of human tumor cells’ impact on human neurons, in contrast to the GBM orthotopic mouse model. Recent studies have similarly employed such models to explore the tumor–neuron interactions during tumor development and progression [14,15]. Moreover, glioblastoma organoid models demonstrate that tumor cells specifically inhibit axon growth in the dorsal brain [11]. In the study presented here, significant neuronal loss was detected in the direct GSC–neuron coculture but not in the indirect coculture, suggesting that cell-to-cell contact between the GSCs and neurons is necessary for the GSCs to induce neuronal death. This is supported by the neuronal toxicity induced by the CM from the GSC–neuron coculture Similar to the in vivo results presented in Figure 1 and Figure 2, radiation did not appear to influence the neuronal death induced by the coculture with the GSCs. These differences could be due to the high density of tumor cells in recurring tumor masses. These findings support reports that state that physical force and a radiation-induced altered tumor microenvironment (a modified ECM and biochemical changes) damage the neurons and brain tissues [33,34,35]. Similarly, there was no significant impact of radiation on neuronal death and the cytokine profile in the cocultures as compared to these properties in the non-irradiated ones, demonstrating that irradiation does not enhance coculture-induced neurotoxicity. To support these findings, we further show that irradiation does not impart additional DNA damage to the neurons in cocultures; however, the GSCs very efficiently repair irradiation-induced DNA damage when in direct contact with the neurons in cocultures. These observations provide an insight into the role of the neurons in mediating the DNA repair capabilities of the GSCs, which could be one of the possible mechanisms underlying tumor recurrence and treatment resistance post-radiotherapy in GBM patients. These are substantial findings that require further investigation to understand the role of radiation in neuronal death in GBM.

IL-8 was secreted into the coculture media exclusively in the GSC–neuron direct cocultures, leading to further investigations into the activation of the signaling pathways and downstream mediators contributing to GSC-induced neurotoxicity. However, further studies are required to understand whether the neurons or GSCs induce IL-8 production in the coculture model and whether this effect is autocrine or paracrine. Also, IL-8-induced neuron death was observed in our IL-8 treatment and supporting IL-8-blocking antibody experiments, supporting the literature indicating that IL-8 regulates GBM plasticity and tumorigenesis [36,37]. Previously, it has also been shown that IL-8 influences the stemness properties of GSCs [38,39]. Therefore, IL-8 contributes to GSC-mediated neuronal death in GBM. However, the coculture CM are more toxic to the neurons than IL-8. These findings suggest that the interaction between the GSCs and neurons produces a complex mix of cytokines that could have an additive effect in inducing neuron death. Moreover, our study highlights the observation that irradiation does not contribute to neurotoxicity, but there is substantial neuronal loss under radiation-induced tumor recurrence in mice. These results are partially supported by studies that have demonstrated that radiation induces alterations in the tumor microenvironment; molecular pathways like mTOR and EGFR signaling; epigenetic modifications in recurring tumor cells; and several physical forces which lead to tumor regrowth with more resilience and aggressiveness, enhancing neurotoxicity and the loss of neuronal networks, leading to neurological disorders in GBM patients [17,24,34,40]. The present study provides insights into the role of GBM tumor cell/GSC interactions with the neurons in tumor recurrence and progression.

The GSC–neuron coculture model used in the present study is simple to use and mimics the biology of GBM in humans; however, it has some limitations, as GSCs have the plasticity to differentiate into neurons, and both GSCs and neurons have overlapping cellular markers [41], which makes it difficult to study certain assays like cell death assays with particular cell types. We used GFP-expressing GSC cells, which was an advantage; however, there are constraints in using a combination of antibodies due to cross-reacting species and fluorochromes. Moreover, neuron cultures are intricately complicated, fragile, and prone to death, which requires extra care and caution.

## 5. Conclusions

In conclusion, both our in vivo and in vitro experimental results suggest that GBM cells induce neuronal death, which could contribute to seizures and epilepsy-mediated neurological disorders in GBM patients.

## Figures and Tables

**Table 1:** The list of antibodies used in this study.

Antibody	Target Species	Species	Isotype	Dilution	Product Catalog	Company
NeuN	Human	Rabbit	IgG	1:1000	ABN78	Sigma
Tuj1 (β Tubulin III)	Human	Mouse	IgG	1:500	MAB1637	Sigma
Anti-phospho histone H2.AX, clone JBW301	Human	Mouse	IgG	1:1000	05-636	Sigma
GFP	Human	Goat	IgG	1:500	AB6673	Abcam (Burlington, MA, USA)
PARP	Human	Rabbit	IgG	1:1000	9542	Cell Signaling (Danvers, MA, USA)
Actin, clone C4	Human	Mouse	IgG	1:1000	MAB1501	Sigma
AF-488	Mouse	Donkey	IgG	1:1000	A21202	Invitrogen
AF-488	Goat	Donkey	IgG	1:1000	A31814	Invitrogen
AF-647	Mouse	Donkey	IgG	1:1000	A32787	Invitrogen
AF-647	Rabbit	Donkey	IgG	1:1000	A21239	Invitrogen
Anti-mouse HRP-linked	Mouse	Donkey	IgG	1:1000	7076S	Invitrogen
Anti-rabbit HRP-linked	Rabbit	Donkey	IgG	1:1000	7074S	Invitrogen
IL-8-neutralizing antibody	Human	Mouse	IgG	0.5 µg/mL	AB18672 (807)	Abcam

This table describes the details of antibodies used in ICC, IHC, and Western blotting.

## Data Availability

The authors declare that the data supporting the findings of the current study are available in this paper and can be made available upon request by the corresponding author.

## Supplementary Materials

The following supporting information can be downloaded at https://www.mdpi.com/article/10.3390/cancers17172817/s1. Figure S1: Radiation-induced DNA damage. Figure S2: Representative membranes from cytokine arrays for each cell type. Figure S3: Cytokine array. Document S1: Original gels and blots.

## Abbreviations

The following abbreviations are used in this manuscript.

GBM	Glioblastoma
GSCs	Glioblastoma stem cells
IL	Interleukin
IHC	Immunohistochemistry
USA	United States of America
CM	Conditioned media
h	Hour
NPCs	Neural progenitor cells
OB	Olfactory bulb
RH	Right hemisphere
CTX	Cerebral cortex
TH	Thalamus
FGF	Fibroblast growth factor
AF	Alexa Fluor
RT	Room temperature
r	Recombinant
MCP	Monocyte chemoattractant protein
