# Supplementary material for: Glioblastoma Cells Induce Neuron Loss In Vivo and In Vitro
Source: Cancers (Basel). 2025 Aug 28;17(17):2817. doi: 10.3390/cancers17172817 (PMC12427526; doi:10.3390/cancers17172817)
Supplement: Supplementary file 1 [file cancers-17-02817-s001.zip › cancers-3803996-Supplementary Document S1.pdf]

cancers

MDPI

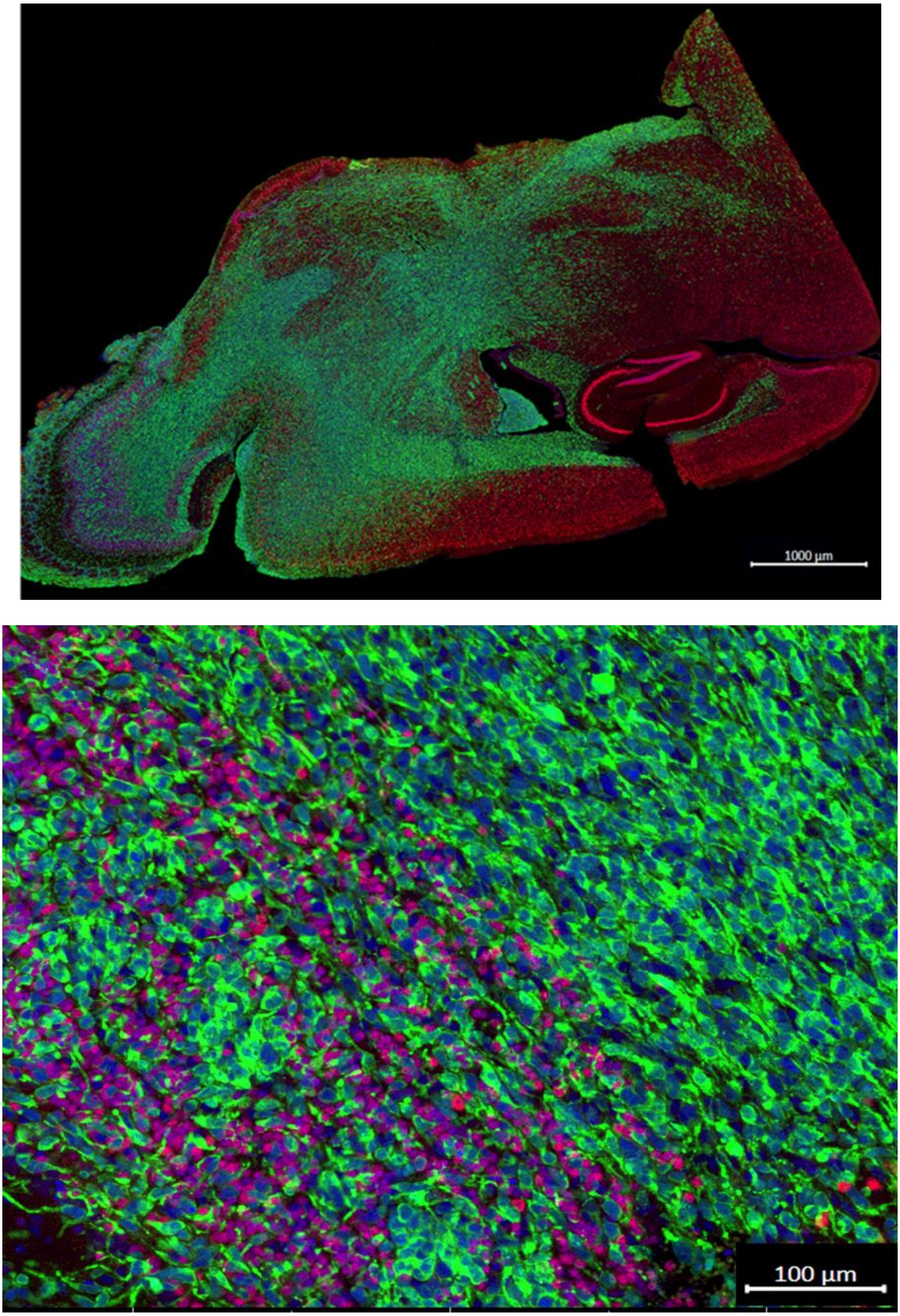

Figure 1. a. 0Gy Tumors − 40× Scan and magnified image (35%).

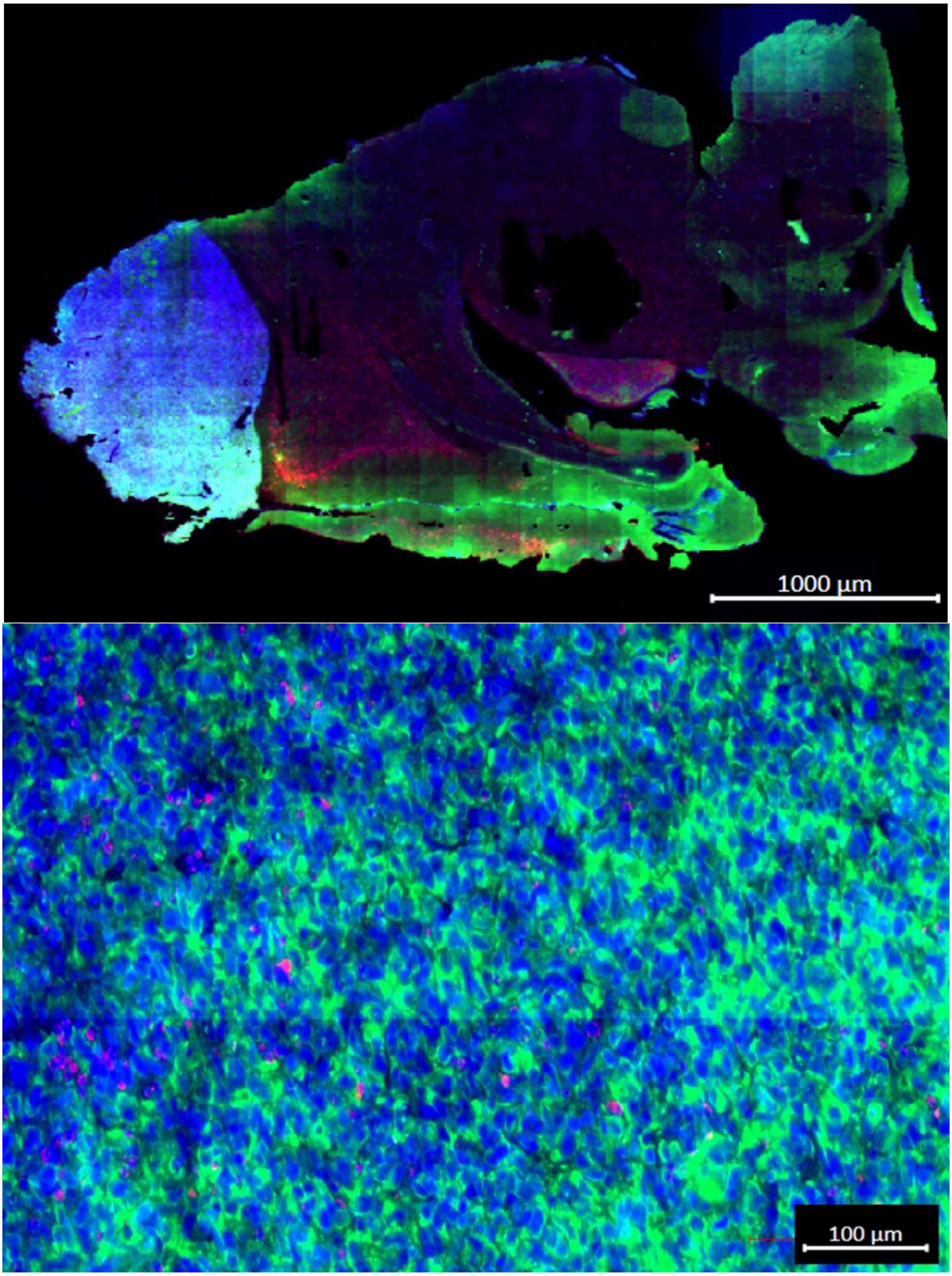

Figure 1. b. 10Gy − 14 days. 40× Scan and magnified image (35%).

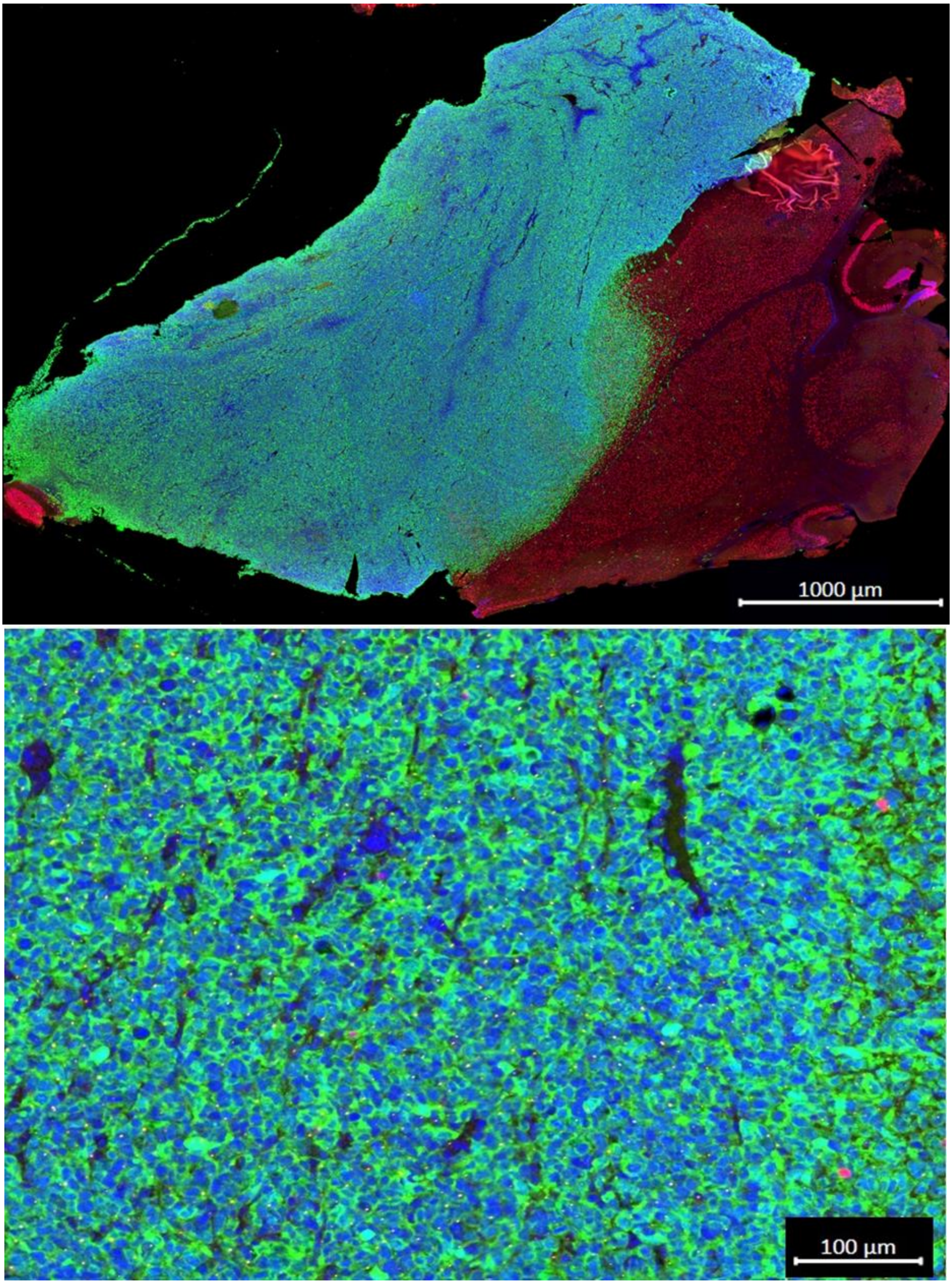

Figure 1. c. 10Gy − 21 Days 40× and magnified image (35%).

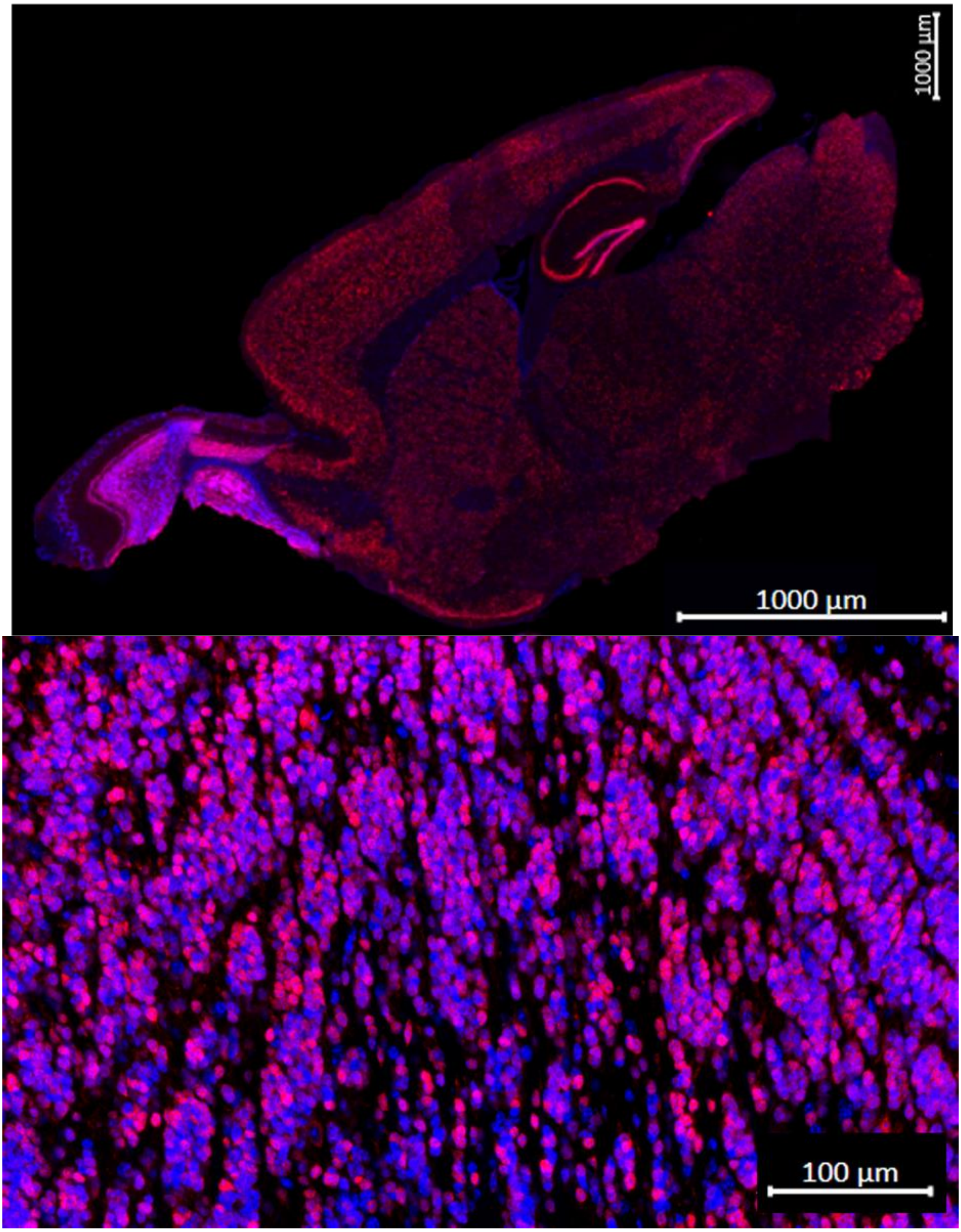

Figure 2. a. 0Gy−40× scan and magnified image (35%).

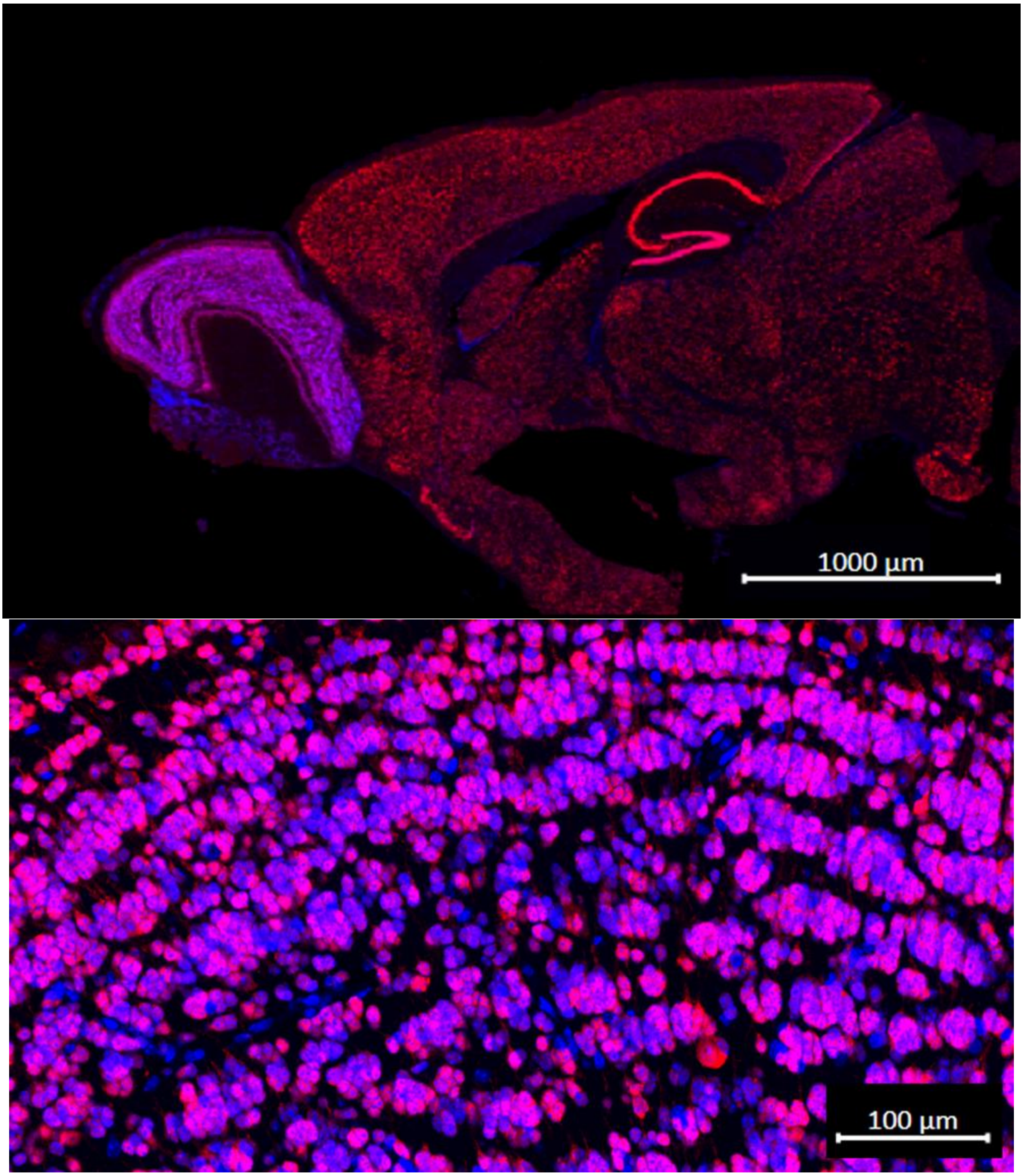

Figure 2. b. 10Gy − 14 day 40× and magnified image (35%).

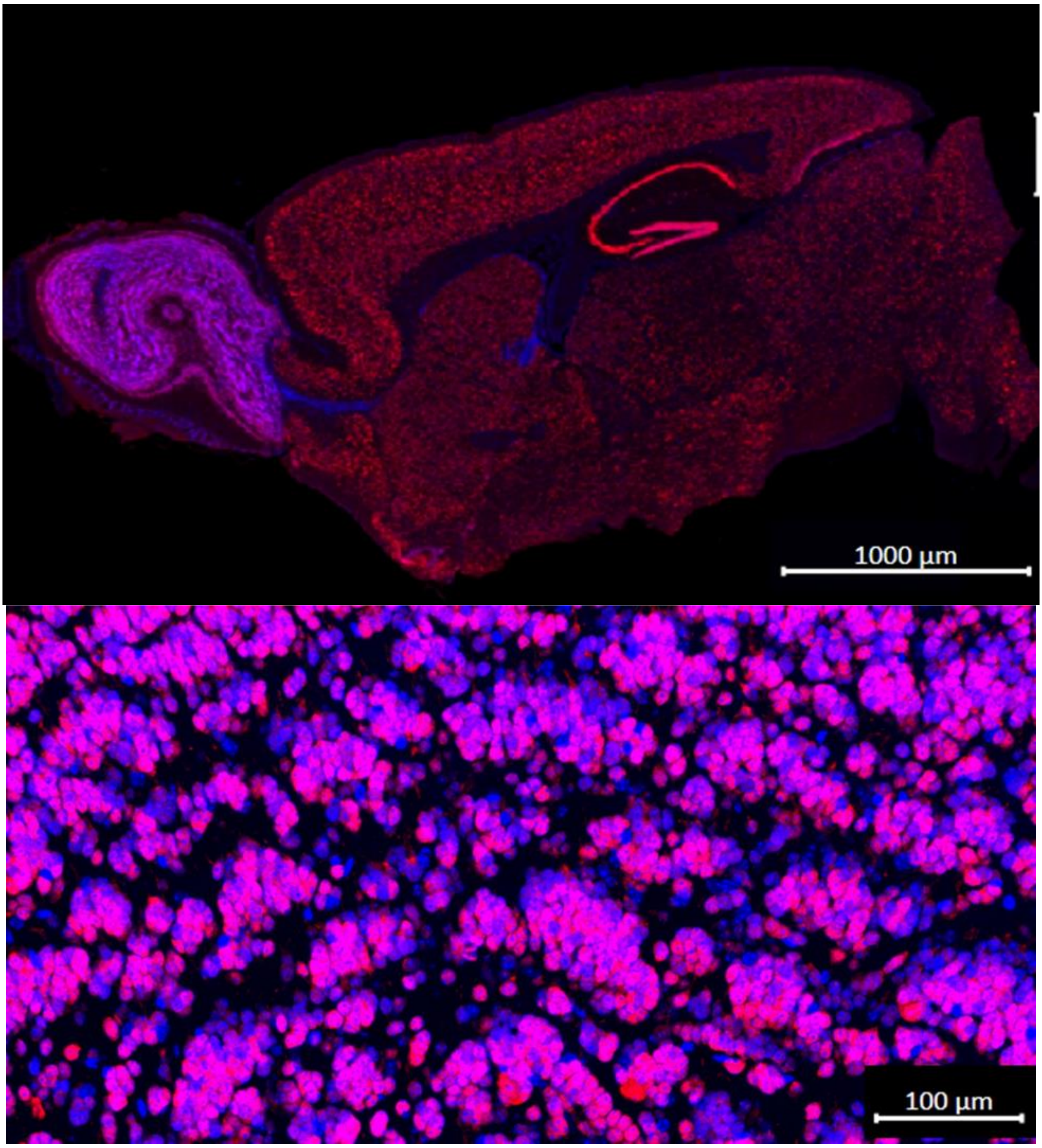

Figure 2. c. 10Gy − 21 Days 40× and magnified image (35%).

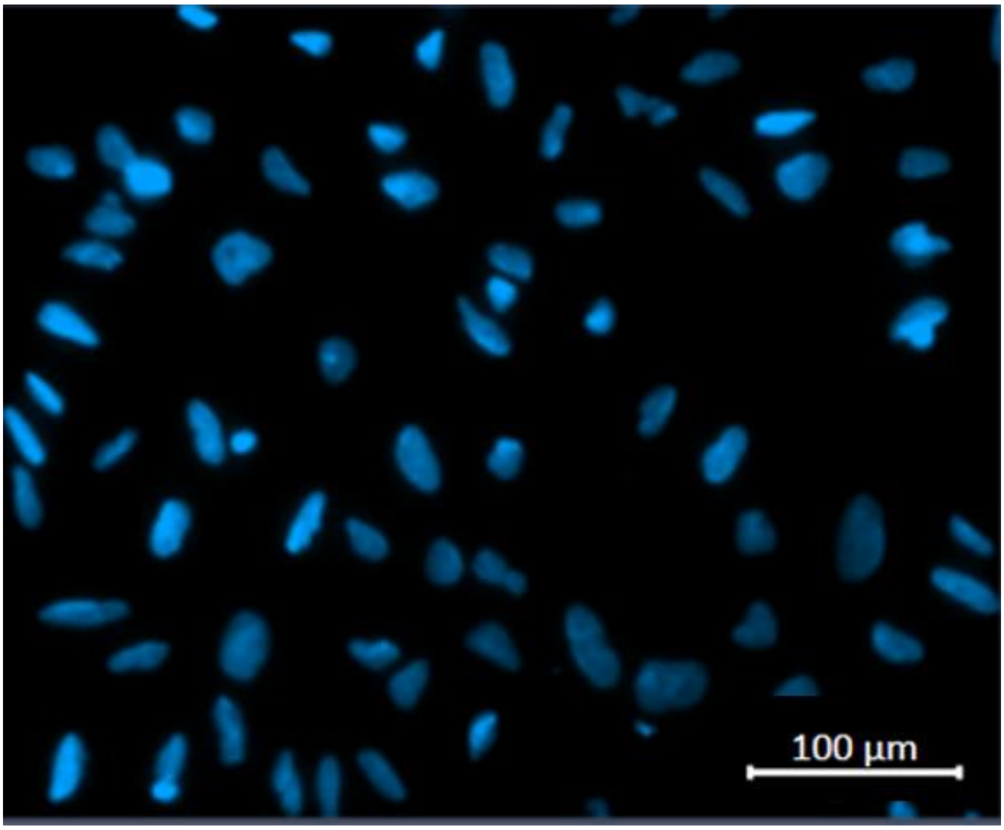

Figure 3. a. NPC DAPI.

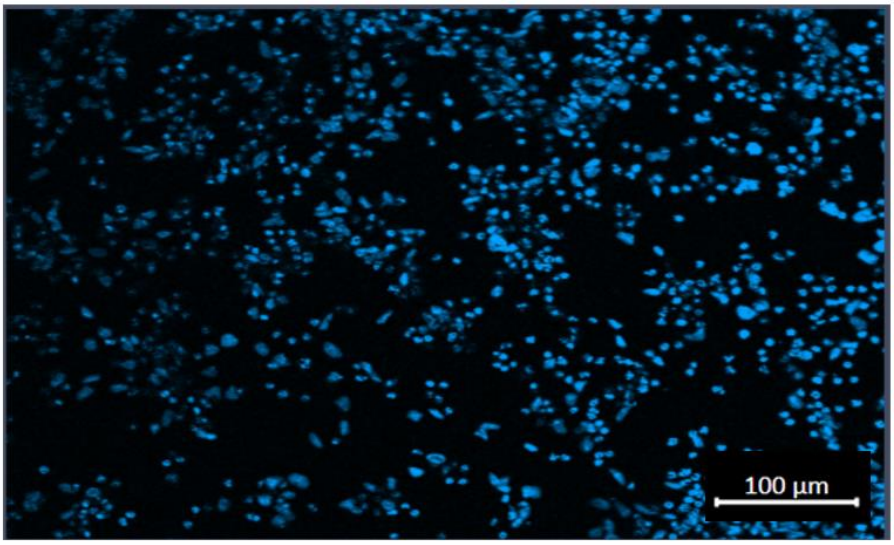

Figure 3. a. Neurons DAPI.

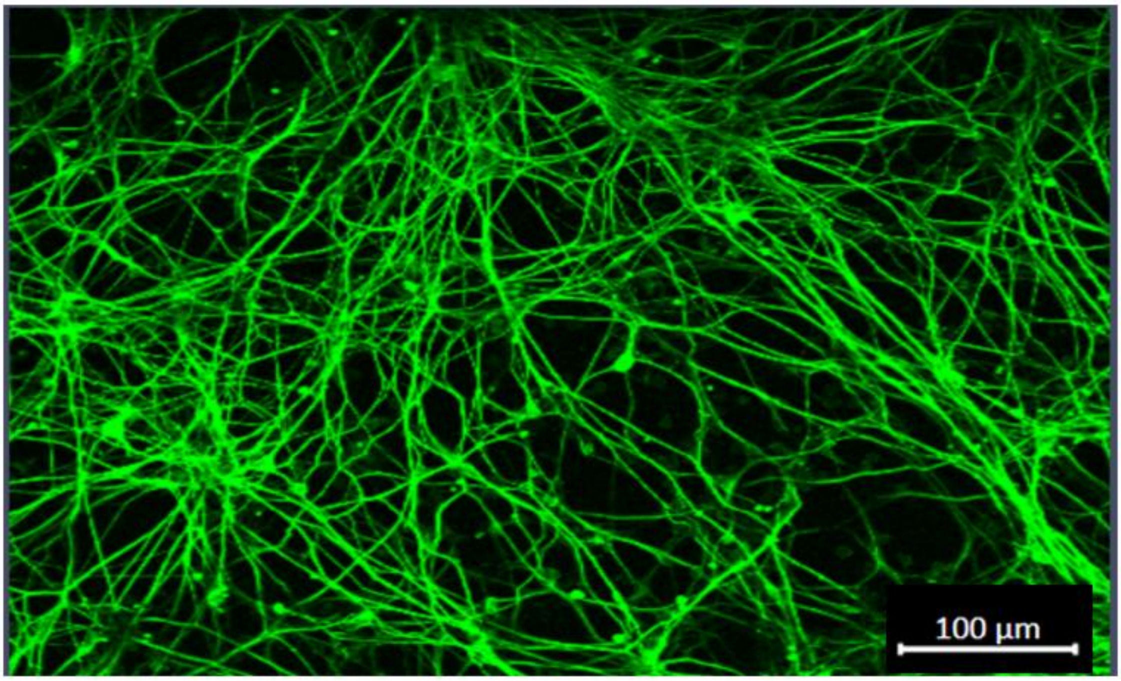

Figure 3. a. Neurons Tuj1.

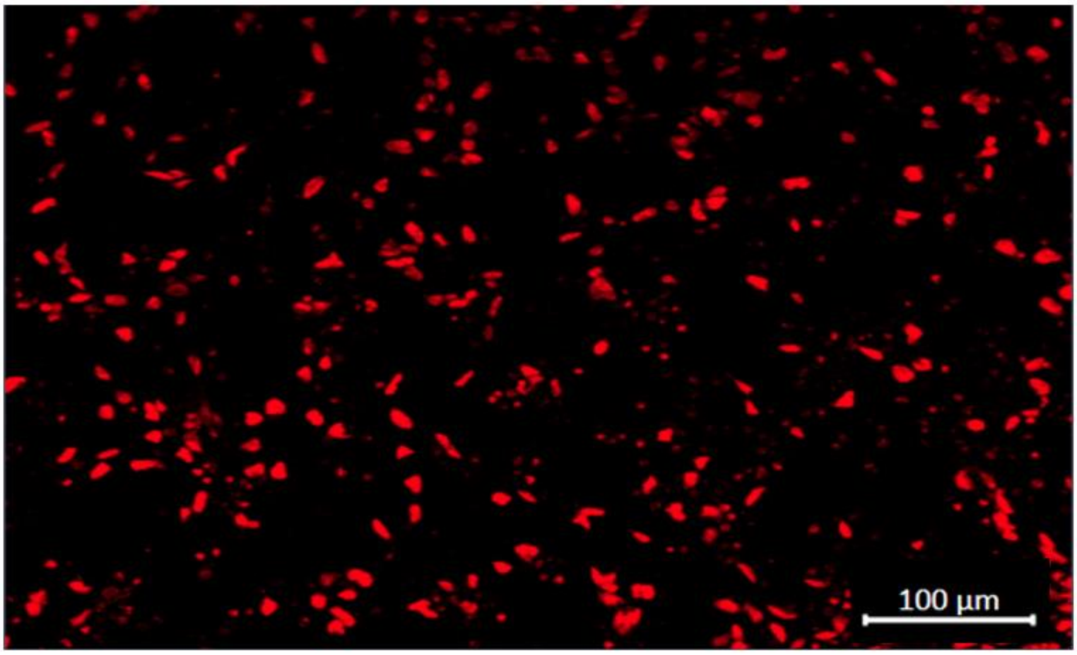

Figure 3. a. Neurons NeuN.

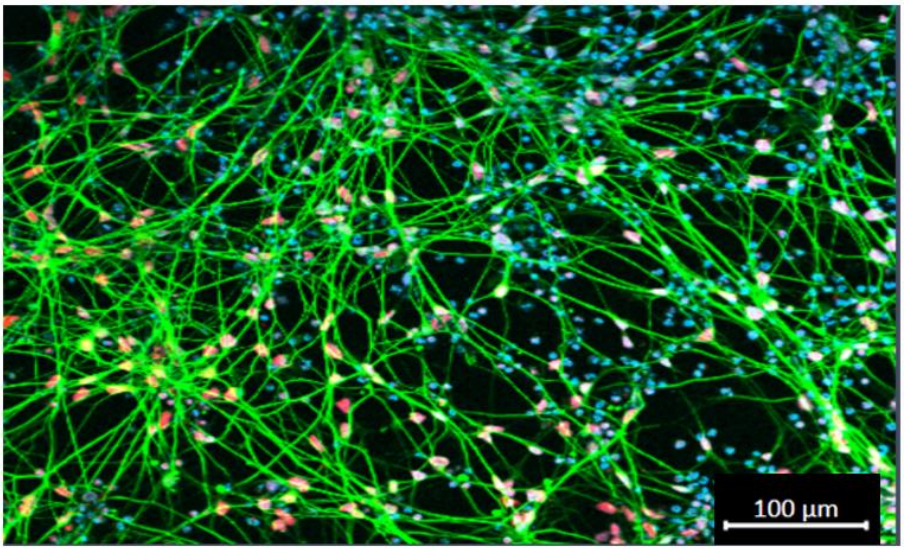

Figure 3. a. Neurons Tuj1 DAPI NeuN merged.

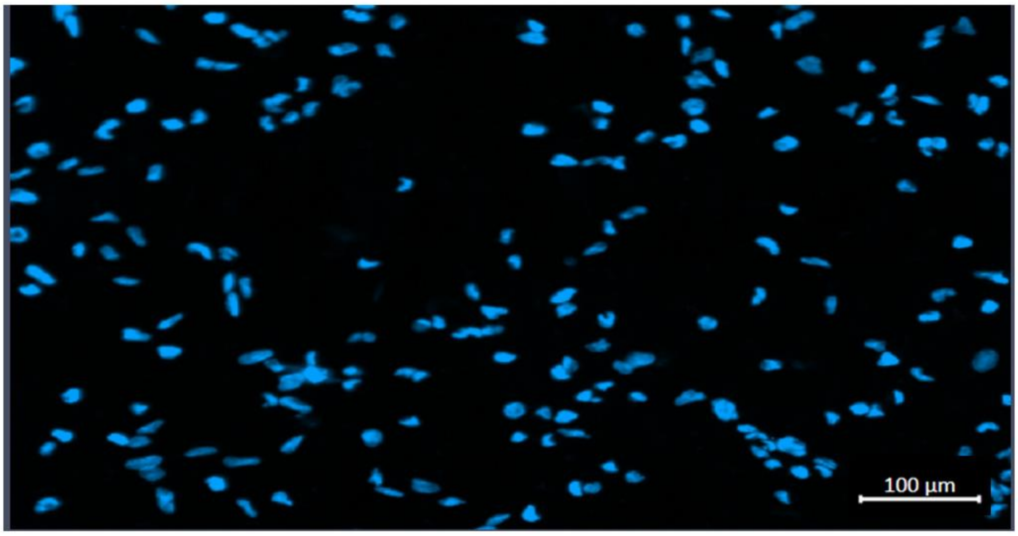

Figure 3. b. GSC DAPI.

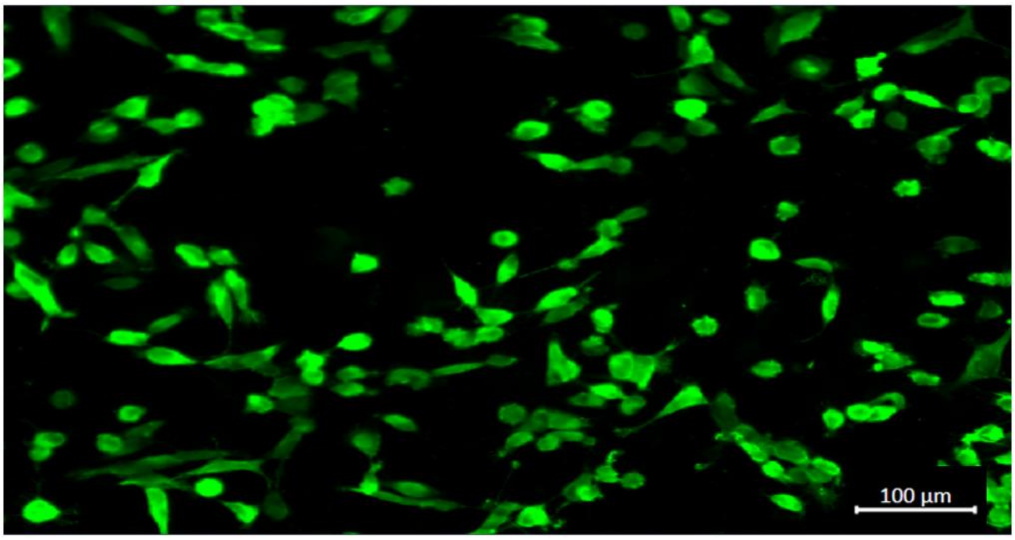

Figure 3. b. GSC GFP.

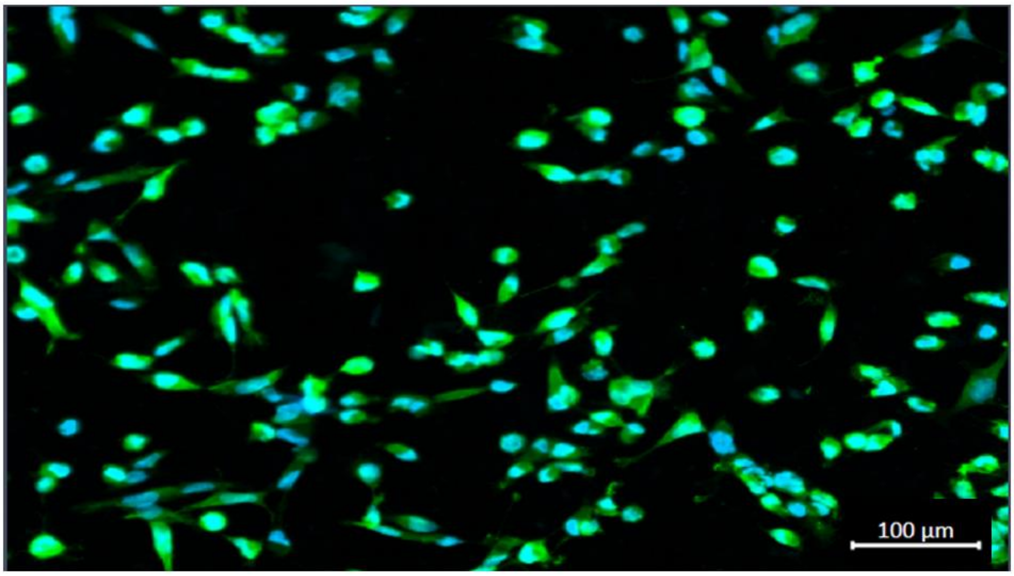

Figure 3. b. GSC Merged.

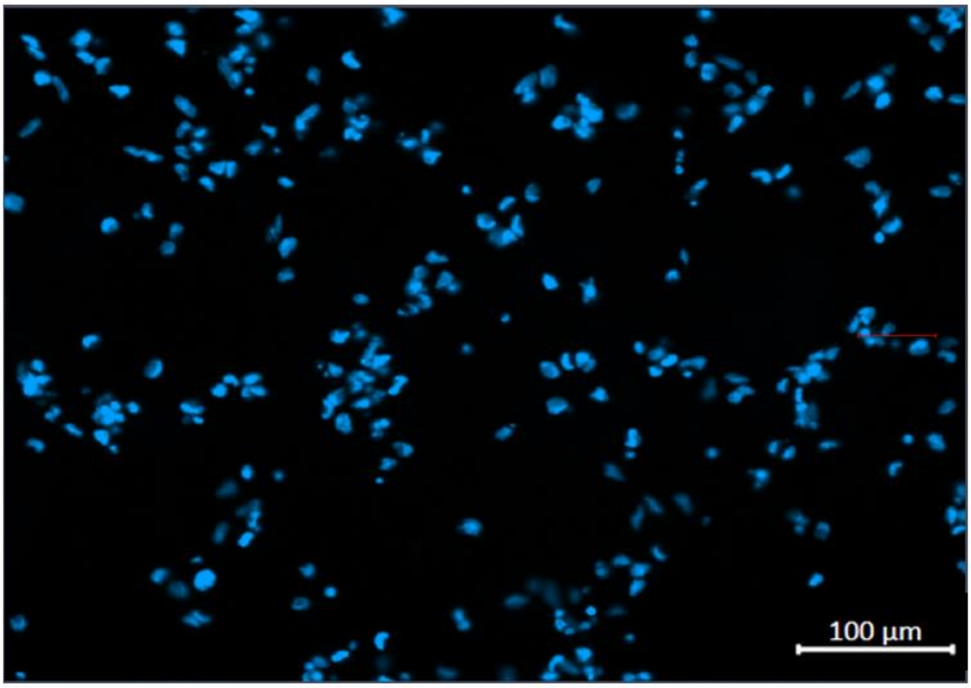

Figure 3. b. Coculture DAPI.

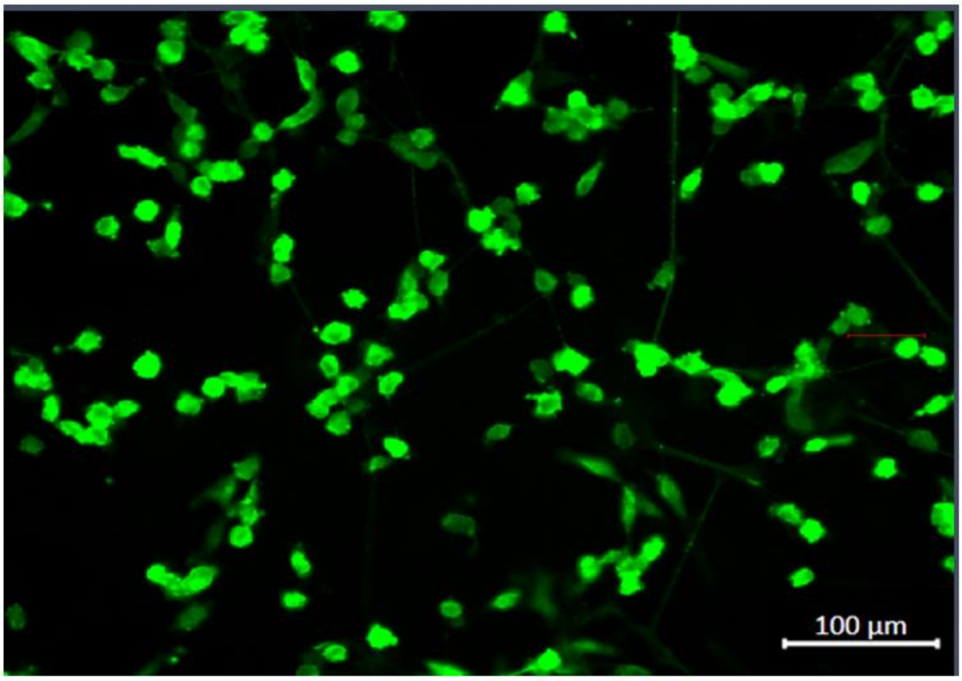

Figure 3. b. Coculture GFP.

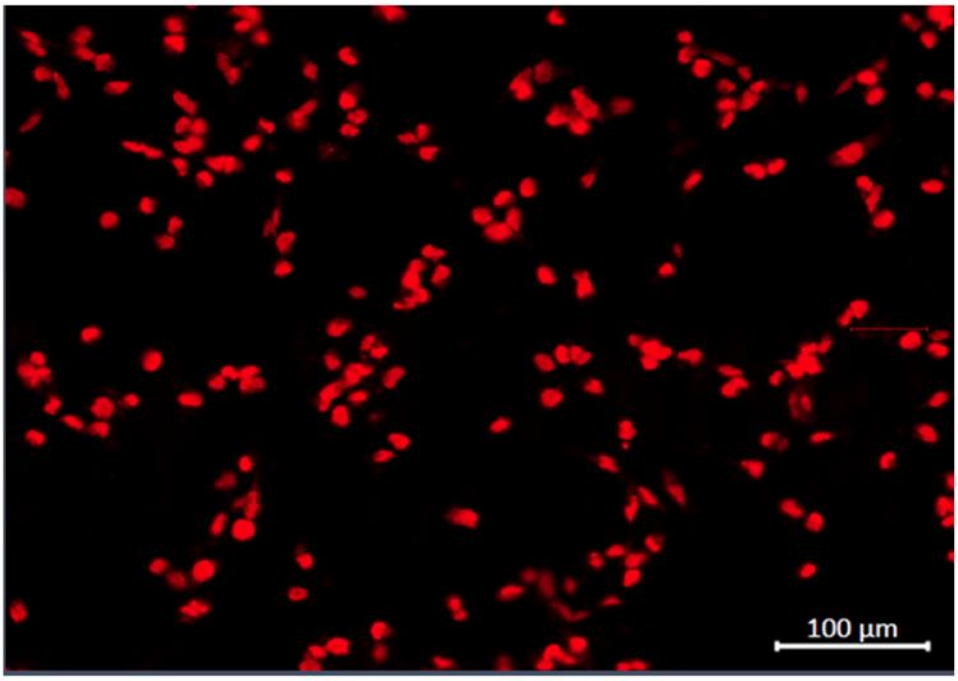

Figure 3. b. Coculture Neun^{+}.

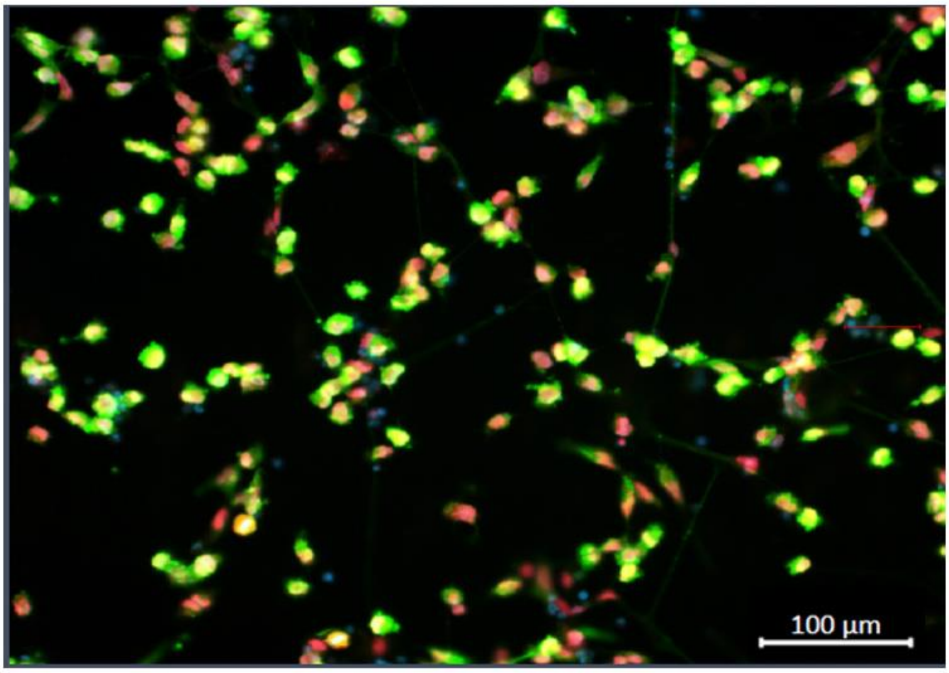

Figure 3. b. Coculture DAPI NeuN GFP merged.

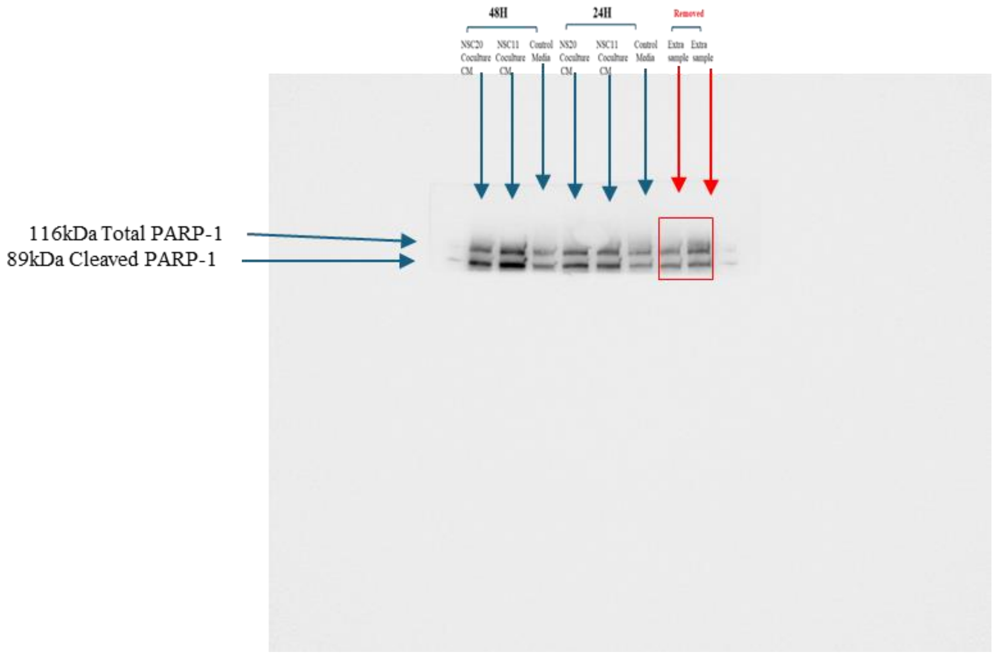

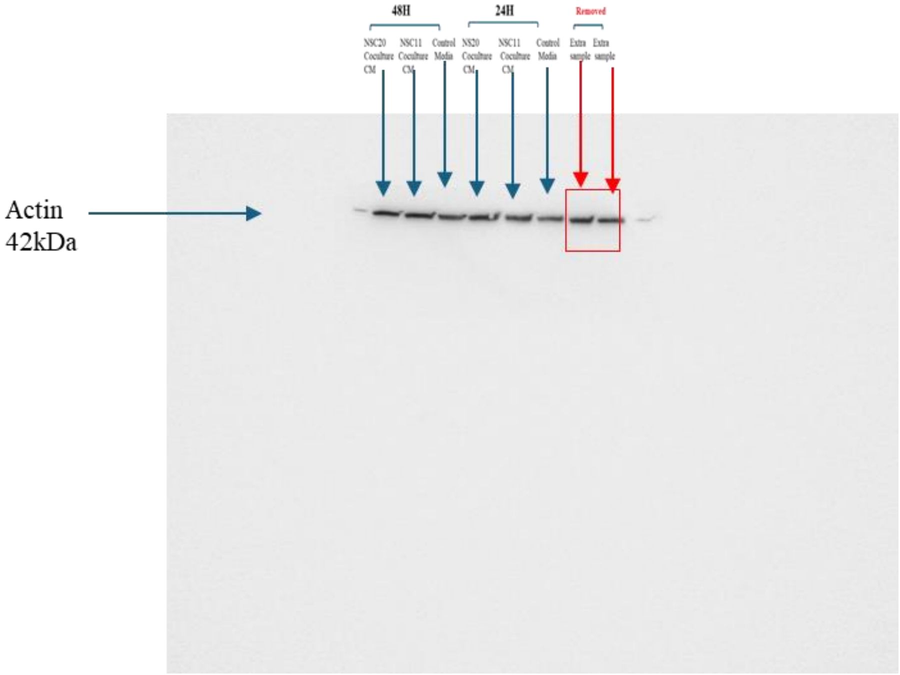

Figure 6. e. Western Blot of PARP – original blot in reverse direction. The last two lanes have extra samples. The image has been horizontally flipped and the two extra samples have been cropped and the image has been presented in the revised manuscript.

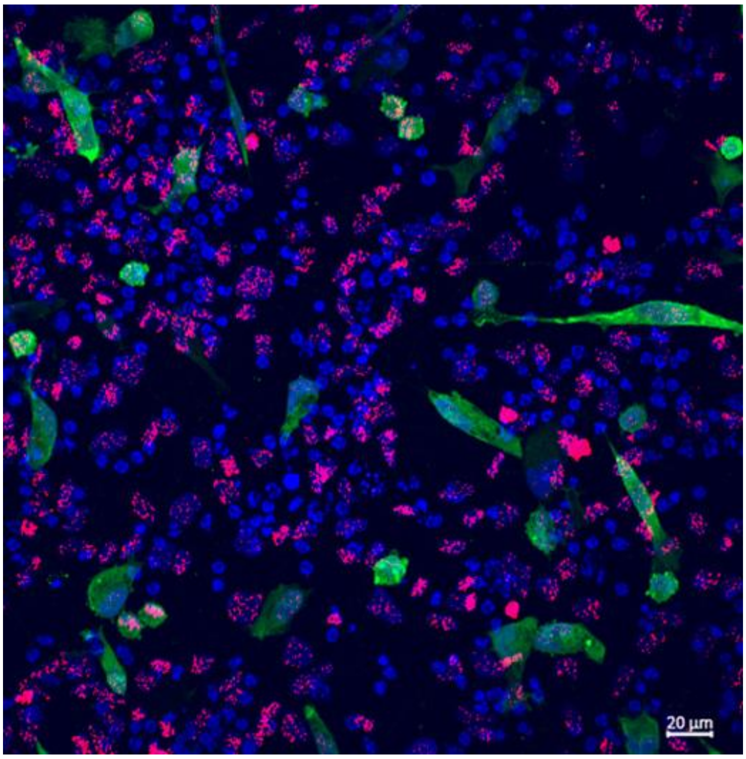

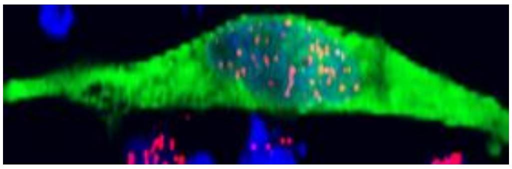

γH2AX foci in GFP expressing GSC

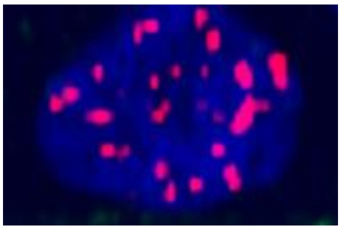

γH2AX foci in neuron cell

Figure S1. a – Representative image of γH2AX foci in GSC-neuron coculture.

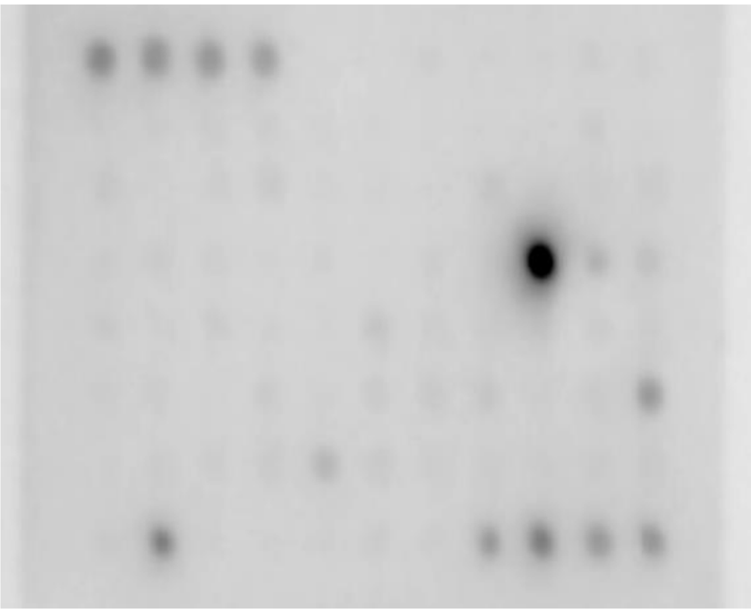

Figure S2. a – Cytokine profile original blot – Neuron CM.

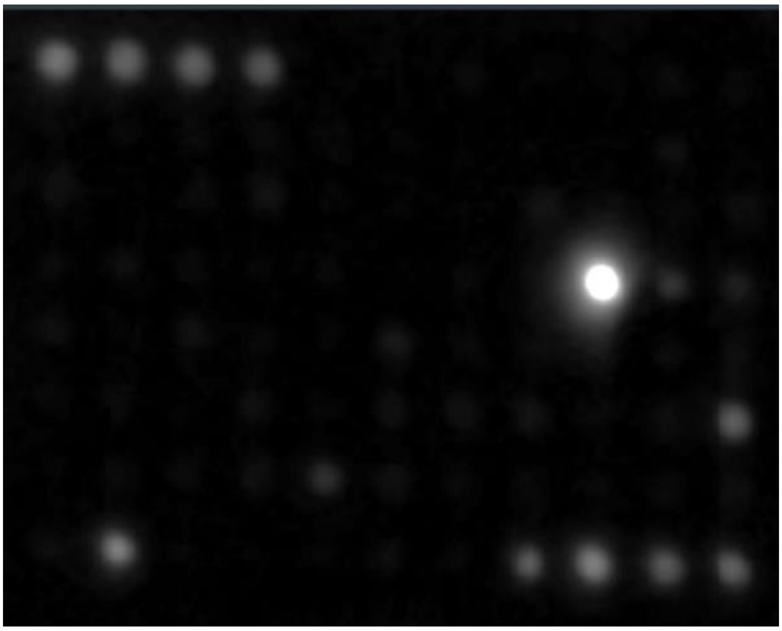

Figure S2. b – Cytokine profile background subtracted blot – Neuron CM.

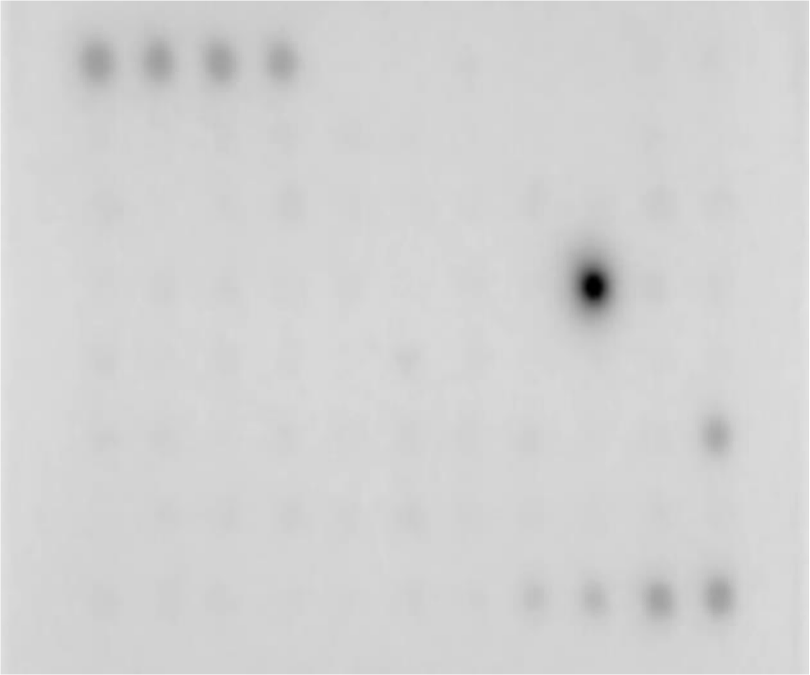

Figure S2. c – Cytokine profile original blot – GSC CM.

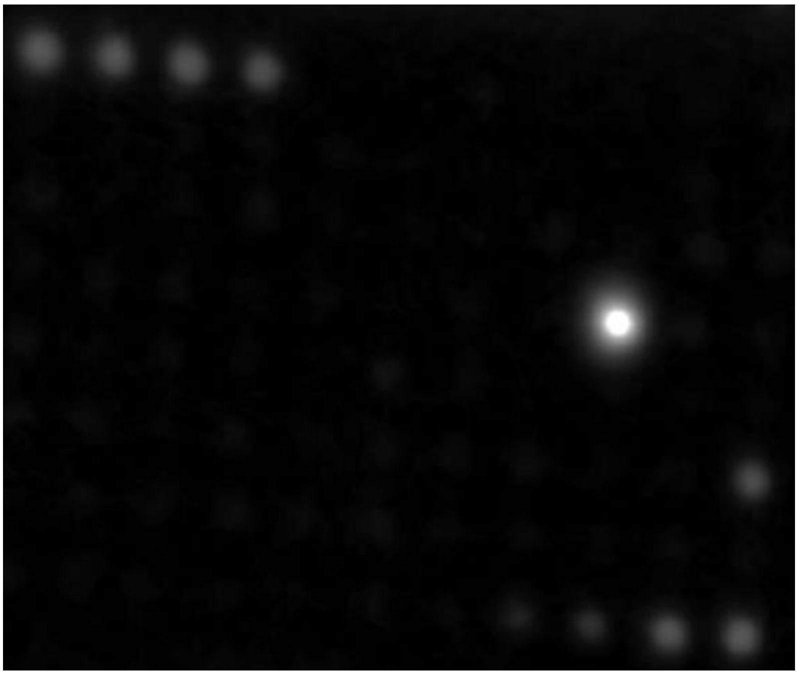

Figure S2. d – Cytokine profile background subtracted blot – GSC CM.

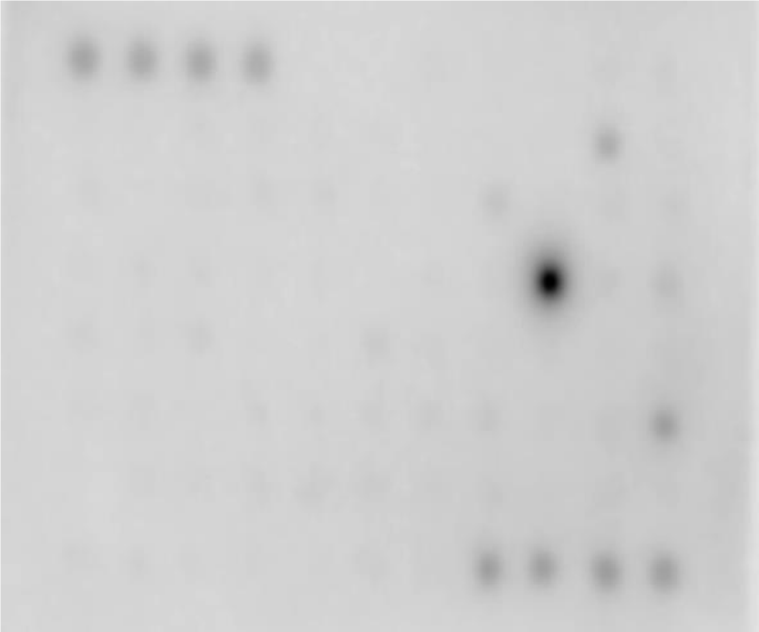

Figure S2. e – Cytokine profile original blot – Coculture CM.

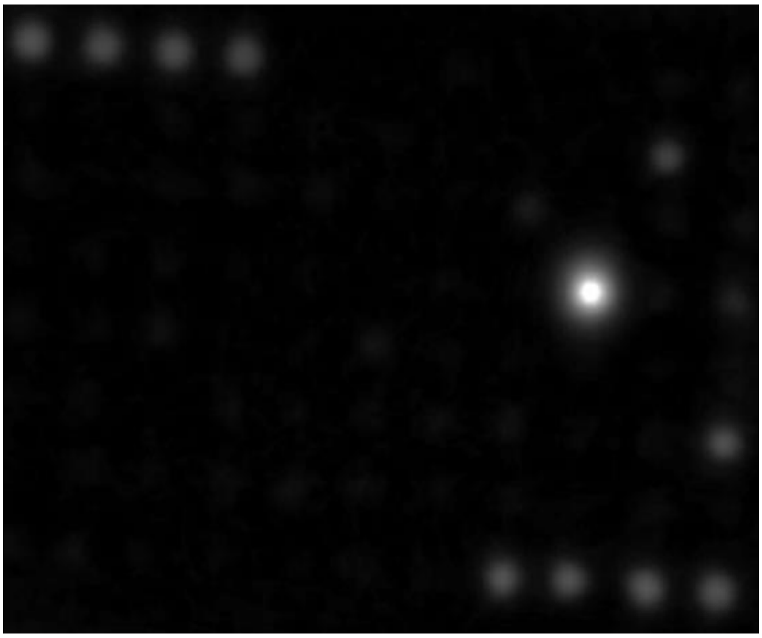

Figure S2. f. Cytokine profile background subtracted blot – Coculture CM.

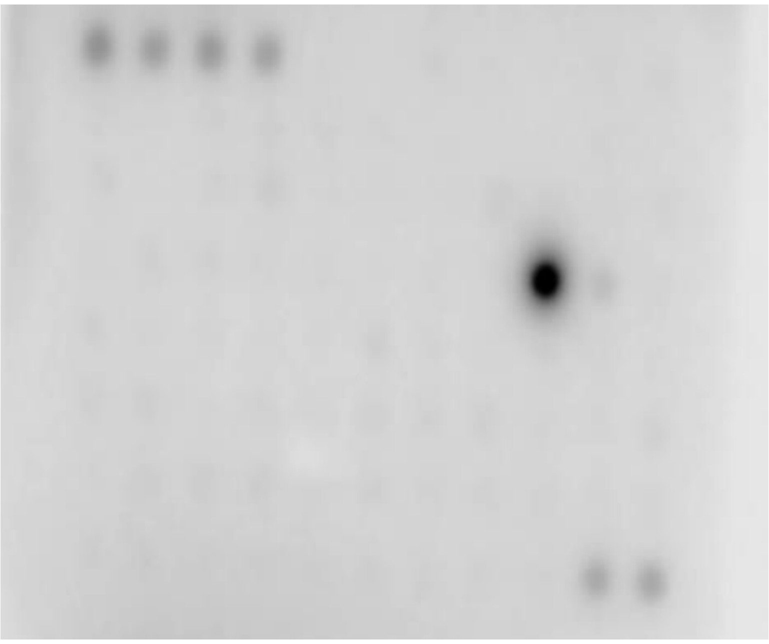

Figure S2. g – Cytokine profile original blot – control GSC Media.

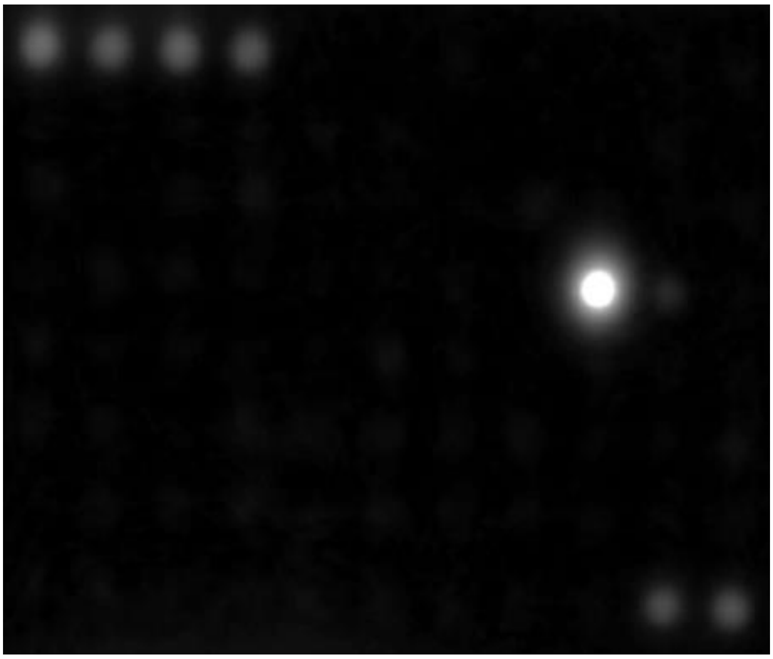

Figure S2. h. Cytokine profile background subtracted blot – control GSC Media.

Disclaimer/Publisher’s Note: The statements, opinions and data contained in all publications are solely those of the individual author(s) and contributor(s) and not of MDPI and/or the editor(s). MDPI and/or the editor(s) disclaim responsibility for any injury to people or property resulting from any ideas, methods, instructions or products referred to in the content.
