# Supplementary material for: Glioblastoma Cells Induce Neuron Loss In Vivo and In Vitro
Source: Cancers (Basel). 2025 Aug 28;17(17):2817. doi: 10.3390/cancers17172817 (PMC12427526; doi:10.3390/cancers17172817)
Supplement: Supplementary file 1 [file cancers-17-02817-s001.zip › cancers-3803996-Supplementary Figure.pdf]

cancers

MDPI

Supplementary Materials: Glioblastoma Cells Induce Neuron Loss in Vivo and in Vitro

Komal N Rawal, Charlotte Degorre and Philip J Tofilon

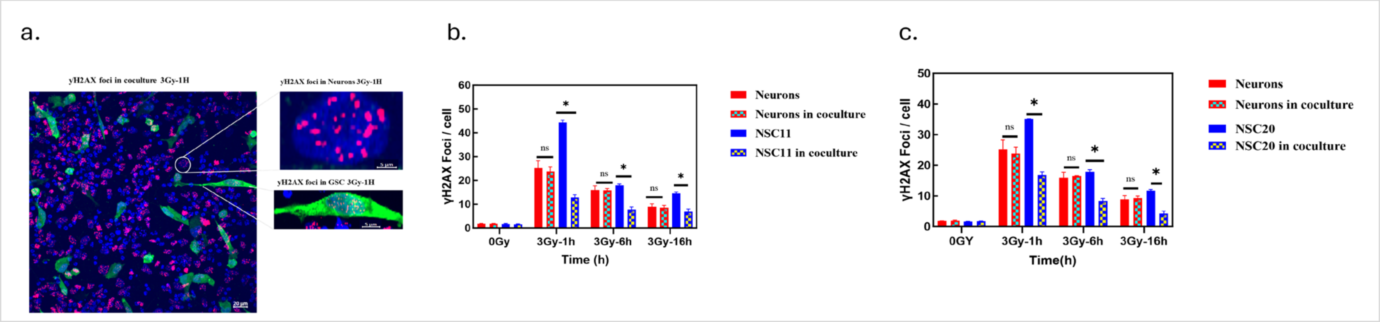

Figure S1. Radiation induced DNA damage: (**a**) Representative image of γH2AX foci in GSC-neuron direct coculture. Scale bar − 20µm. For simplicity neurons are stained with DAPI (blue), GSC are stained with GFP (green) and γH2AX foci appear in red. The magnified image is of a neuron cell with DAPI and γH2AX foci. Scale bar − 5um. The other image is of GSC showing GFP and γH2AX foci. Scale bar − 5um. (**b**) γH2AX foci of NSC11-neurons after 3Gy. The results are presented as Mean of γH2AX foci/cell ± S.E.M. of three independent experiments. *p* value * < 0.05. , *p* value ns > 0.05 (non- significant) (**c**) γH2AX foci of NSC20 − neurons after 3Gy. The results are presented as Mean of γH2AX foci/cell ± S.E.M. of three independent experiments. *p* value * < 0.05. , *p* valu ns > 0.05 (non- significant)

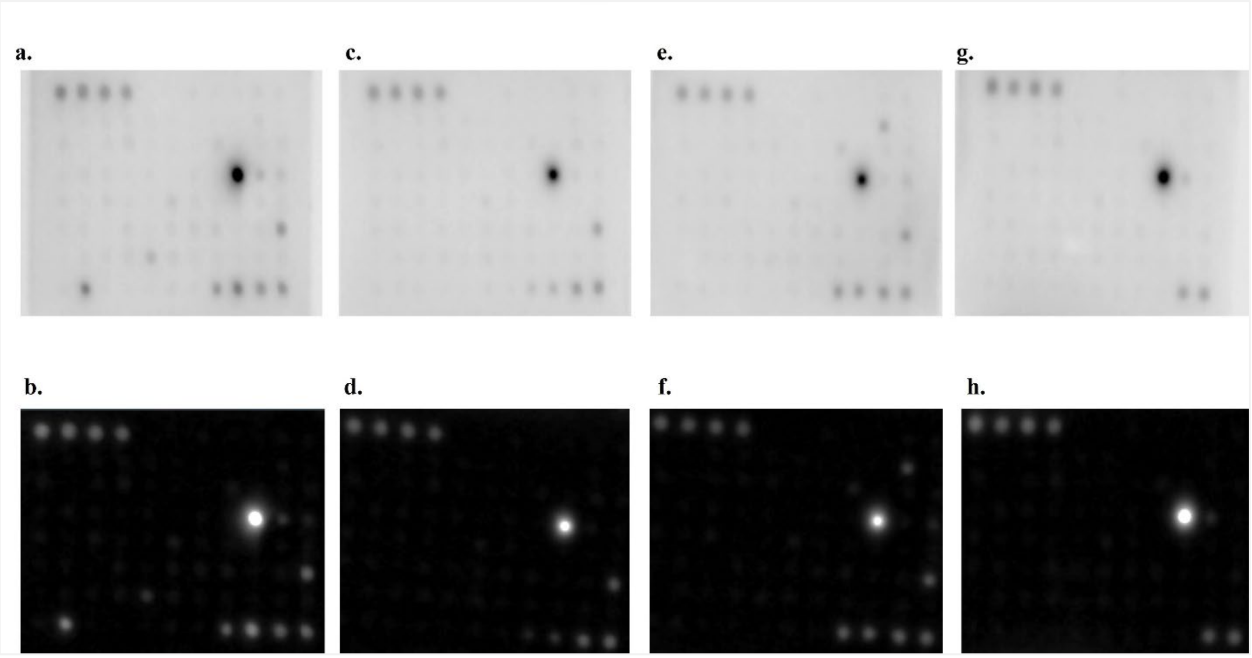

Figure S2. Cytokine Array representative membrane for each cell type (**a**) Original membrane of Neuron CM (**b**) Background subtracted membrane of Neuron CM (**c**) Original membrane of GSC CM (**d**) Background subtracted membrane of GSC CM (**e**) Original membrane of GSC-neuron coculture CM (**f**) Background subtracted membrane of GSC-neuron coculture CM (**g**) Original membrane of GSC control media (**h**) Background subtracted membrane of GSC control media.

 https://doi.org/10.3390/cancers17172817

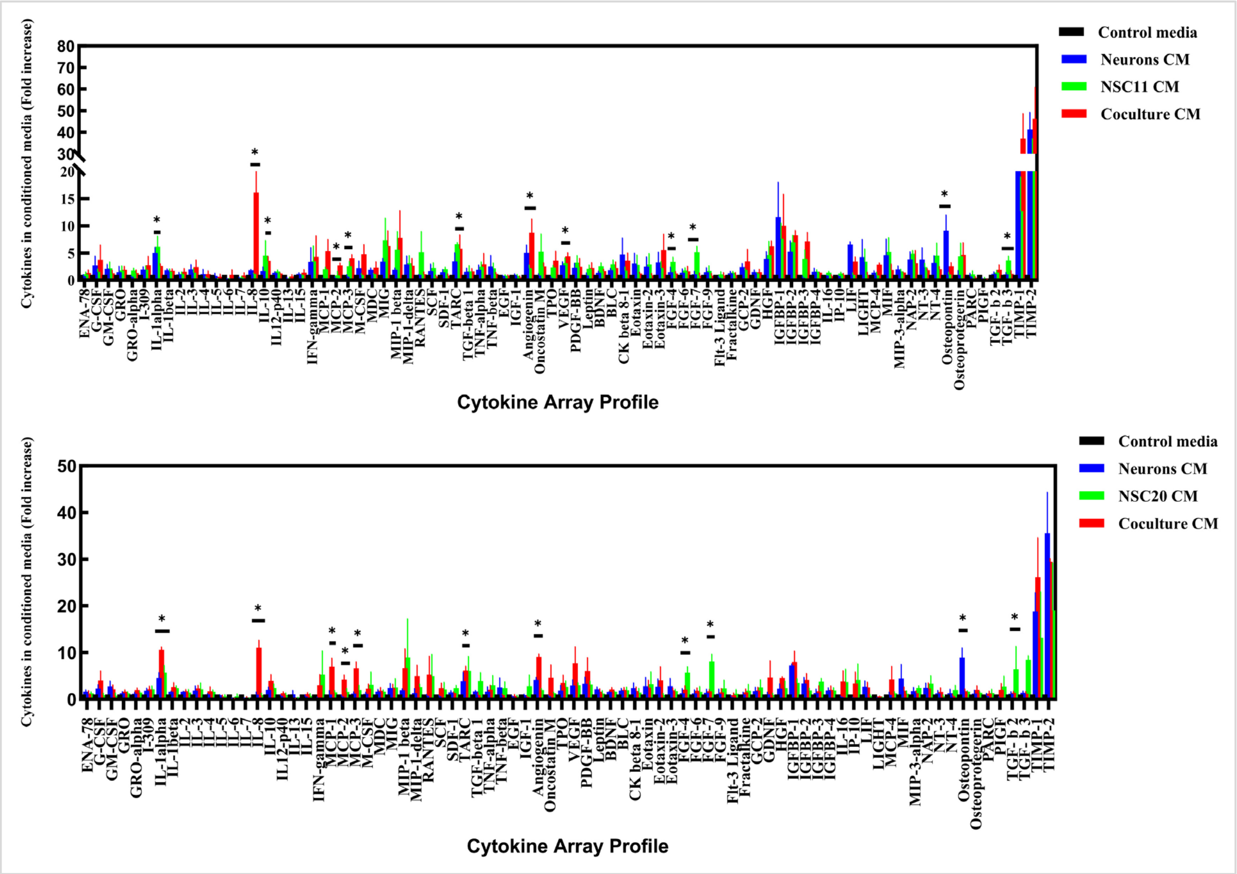

Figure S3. Cytokine Array (**a**) Cytokine Array of 80 cytokines presented as Mean Fold change ± S.E.M. from NSC11-neuron coculture CM normalized to control stem cell media of three independent experiments, *p* value <0.05. (**b**) Cytokine Array of CM generated from NSC20-neuron coculture represented as Mean Fold change ± S.E.M., *p* value <0.05.

Disclaimer/Publisher's Note: The statements, opinions and data contained in all publications are solely those of the individual author(s) and contributor(s) and not of MDPI and/or the editor(s). MDPI and/or the editor(s) disclaim responsibility for any injury to people or property resulting from any ideas, methods, instructions or products referred to in the content.
